# Analysis of *Mycobacterium tuberculosis* Genotypic Lineage Distribution in Chile and Neighboring Countries

**DOI:** 10.1371/journal.pone.0160434

**Published:** 2016-08-12

**Authors:** Jaime Lagos, David Couvin, Loredana Arata, Javier Tognarelli, Carolina Aguayo, Tamara Leiva, Fabiola Arias, Juan Carlos Hormazabal, Nalin Rastogi, Jorge Fernández

**Affiliations:** 1 Subdepartment of Molecular Genetics, Public Health Institute of Chile, Santiago, Chile; 2 WHO Supranational TB Reference Laboratory, TB and Mycobacteria Unit, Institut Pasteur de la Guadeloupe, Guadeloupe, France; 3 Mycobacteria Laboratory, Public Health Institute of Chile, Santiago, Chile; 4 Subdepartment of Infectious Disease, Public Health Institute of Chile, Santiago, Chile; University of Padova, Medical School, ITALY

## Abstract

Tuberculosis (TB), caused by the pathogen *Mycobacterium tuberculosis* (MTB), remains a disease of high importance to global public health. Studies into the population structure of MTB have become vital to monitoring possible outbreaks and also to develop strategies regarding disease control. Although Chile has a low incidence of MTB, the current rates of migration have the potential to change this scenario. We collected and analyzed a total of 458 *M*. *tuberculosis* isolates (1 isolate per patient) originating from all 15 regions of Chile. The isolates were genotyped using the spoligotyping method and the data obtained were analyzed and compared with the SITVIT2 database. A total of 169 different patterns were identified, of which, 119 patterns (408 strains) corresponded to Spoligotype International Types (SITs) and 50 patterns corresponded to orphan strains. The most abundantly represented SITs/lineages were: SIT53/T1 (11.57%), SIT33/LAM3 (9.6%), SIT42/LAM9 (9.39%), SIT50/H3 (5.9%), SIT37/T3 (5%); analysis of the spoligotyping minimum spanning tree as well as spoligoforest were suggestive of a recent expansion of SIT42, SIT50 and SIT37; all of which potentially evolved from SIT53. The most abundantly represented lineages were LAM (40.6%), T (34.1%) and Haarlem (13.5%). LAM was more prevalent in the Santiago (43.6%) and Concepción (44.1%) isolates, rather than the Iquique (29.4%) strains. The proportion of X lineage was appreciably higher in Iquique and Concepción (11.7% in both) as compared to Santiago (1.6%). Global analysis of MTB lineage distribution in Chile versus neighboring countries showed that evolutionary recent lineages (LAM, T and Haarlem) accounted together for 88.2% of isolates in Chile, a pattern which mirrored MTB lineage distribution in neighboring countries (n = 7378 isolates recorded in SITVIT2 database for Peru, Brazil, Paraguay, and Argentina; and published studies), highlighting epidemiological advantage of Euro-American lineages in this region. Finally, we also observed exclusive emergence of patterns SIT4014/X1 and SIT4015 (unknown lineage signature) that have hitherto been found exclusively in Chile, indicating that conditions specific to Chile, along with the unique genetic makeup of the Chilean population, might have allowed for a possible co-evolution leading to the success of these emerging genotypes.

## Introduction

*Mycobacterium tuberculosis* (MTB), a Gram-positive bacterium, is the causative agent for tuberculosis (TB); a disease that presents millions of new cases and deaths every year and is considered as a major threat to public health [1]. Although a quick and assertive diagnosis is essential for early treatment and disease management, many people lack proper access to the facilities required for adequate or early diagnosis. In this context, the molecular typing of *MTB* has greatly improved our knowledge and control of tuberculosis by allowing for: (i) the detection of unsuspected transmission, (ii) the identification of false-positive cultures, (iii) discrimination between reinfection and relapse [2], and (iv) the study and analysis of global biodiversity and phylogeographical variations of the tubercle bacilli [3]. In the last 2 decades, the cumbersome “gold standard” IS*6110*-RFLP methodology necessitating Southern blotting [4,5] was progressively replaced by PCR-based methods, namely spoligotyping and Mycobacterial interspersed repetitive unit-variable number of tandem-repeat (MIRU-VNTR) typing [6–10]. These methods showed satisfactory discriminatory power and reproducibility [9], particularly when used concomitantly that further allows to identify MTB genetic lineages–a prerequisite to study TB population structure at local, regional, and global scale [11].

As far as TB in the Americas is concerned, it accounted for approximately 285,200 new cases of TB in 2013, which equals to 29 new cases per 100,000 inhabitants [12]. Despite two-thirds (69%) of all cases reported being from South American patients, wide epidemiological differences including varying incidence rates were found both country and region wise, highest incidence rates being from Brazil (46/100,000), Bolivia (123/100,000) and Peru (124/100,000) [12]. In Chile different rates of incidence ranging from 7.5/100,000 to 23.7/100,000, were observed, the highest rates being reported from the northern regions of the country [13]. According to the latest available data, the TB incidence in Chile for 2014 was 12.3 per 100,000, which remains significantly higher than the rate of elimination recommended for declassifying TB as a public health problem (<5 per 100,000).

Investigations conducted on MTB genotypic lineage distribution in Southern America have shown that although the Euro-American lineage is the most widely represented, regional differences in the distribution of lineages/sublineages are frequently observed both between as well as within countries [14–22]. According to the SITVIT2, the LAM, Haarlem and T families are the most commonly observed members of the Euro-American lineage in South and Central America and the Caribbean, a distribution profile shared with Europe and Middle Africa [23]. Nonetheless, some regional specificities such as cases involving a LAM family strain designated as RD^Rio^ associated with multiple drug resistance (MDR) in Brazil [18], and the almost exclusive “significant” presence of the Beijing family in Peru and Colombia associated both with MDR as well as XDR cases [20,21] may be cited. It is today possible to pinpoint such specificities as well underline the finer differences in MTB population structure at local, regional and macro regional levels, thanks to international genotyping databases and web tools for molecular epidemiology of tuberculosis [23, 24].

Limited MTB genotyping studies in Chile, conducted primarily in metropolitan regions, suggested that Chile is dominated by three major MTB lineages, LAM, T, and Haarlem [25–27]. To provide with a more comprehensive snapshot of MTB lineages circulating in Chile, we collected and analyzed a total of 458 *M*. *tuberculosis* isolates (1 isolate per patient) originating from all 15 regions of Chile, including the Metropolitan Region and large cities in north (e.g., Iquique) as well as the south (e.g., Concepción). The data on MTB lineage distribution in Chile versus neighboring countries were compared using the SITVIT2 database [3, 28], so as to highlight exclusive emergence of certain genotypes as well as the potential associated risks in Chile.

## Materials and Methods

### Sample collection

A total of 458 isolates (1 isolate per patient) of *M*. *tuberculosis* were collected from 15 regions of Chile (**S1 Fig**). The isolates, obtained from treatment virgin patients (VT), were collected between 2011 (n = 338 or 73.80%) and 2012 (n = 120 or 26.20%), all the isolates were used in the study. Of them, 304 isolates were from male patients (66.38%) and 154 from female patients (33.62%). The isolates were extracted from body fluids (sputum: 95%, bronchoalveolar lavage fluid: 3.7%, tissue: 0.9%, blood 0.2% and pleural fluid: 0.2%). TB was diagnosed in the clinical centers of the respective cities and the diagnosis was confirmed at the Supranational Reference Mycobacteria Laboratory at the Institute of Public Health of Chile which also conducted first and second line drugs susceptibility tests using the Lowenstein–Jensen medium and the proportion method [29].

The strains used in this study were collected routinely during activities of the state TB control program. No patients were contacted to request additional information. The study was reviewed and approved by the review board of Biomedical Department of Public Health Institute who granted permission for use of the MTB isolates and clinical data for the purposes of the study and waived the need for written informed consent from participants. This study is part of the monitoring carried out by the Institute of Public Health of Chile for the diagnosis and characterization of infectious agents.

### DNA extraction and genotyping

MTB isolates obtained from the Lowenstein Jensen medium cultures were resuspended in 500 μL TE (10 mM Tris pH 8.0, 1 mM EDTA pH 8.0). Suspensions were inactivated by heating at 95°C for 15 minutes. Bacterial DNA was isolated by treatment with cetyltrimethylammonium bromide (CTAB) in the presence of 0.7 M NaCl as described previously [30].

Spoligotyping was performed at the Molecular Genetics sub-department of Institute of Public Health of Chile by using the Spoligotyping kit (Ocimum Biosolutions) as described by Kamerbeek *et al*. [8]. Briefly, the 43 spacers between the direct repeats in the target region were amplified using biotinylated primers and the PCR products were then hybridized to a membrane and visualized by chemiluminescence. *M*. *tuberculosis* H37Rv and *M*. *bovis* BCG controls, included in the kit, were used as controls for each run. Spoligotyping results were converted into the octal code for comparison with the SITVIT2 proprietary database of Institut Pasteur de la Guadeloupe which is an updated version of the previously released SITVITWEB database (available at: http://www.pasteur-guadeloupe.fr:8081/SITVIT_ONLINE/) [23]. Within this database, a SIT is created when 2 or more isolates share an identical spoligotyping pattern.

### Phylogenetic and statistical analysis

In order to study the phylogenetic relationships of given spoligotypes obtained in this study (n = 458 isolates), a minimum spanning tree (MST) was created using the BioNumerics version 6.6 software (Applied Maths, Sint-Martens-Latem, Belgium; available at: http://www.applied-maths.com/bionumerics). Furthermore, a spoligoforest tree was constructed using the Fruchterman-Reingold algorithm in the SpolTools software (http://www.emi.unsw.edu.au/spolTools) [31]; the tree was reshaped and colored using the GraphViz software (http://www.graphviz.org) [32]. Unlike the MST, the spoligoforest is a directed graph which tentatively highlights parent to descendant relationships between spoligotypes. Lastly, Fisher’s Exact and Pearson's Chi-squared tests were calculated using R software. A p-value<0.05 was considered statistically significant.

## Results

The present study identified spoligotypes for clinical isolates of *M*. *tuberculosis* (n = 458) with a global male/female sex ratio of 304/154 = 1.97. The isolates were collected from different cities of Chile between the years 2011 and 2012. These cities were: Santiago (n = 181), Concepción (n = 34), Iquique (n = 34), Valparaiso (n = 29), Rancagua (n = 20), Temuco (n = 18), Los Angeles (n = 16), Osorno (n = 15), Arica (n = 14), Puerto Montt (n = 12), Talca (n = 12), Talcahuano (n = 12), Antofagasta (n = 11), Coquimbo (n = 11), Castro (n = 9), Punta Arenas (n = 7), Ovalle (n = 6), Valdivia (n = 6), Copiapo (n = 5), Coihaique (n = 2), Viña del Mar (n = 2), Vallenar (n = 1), and Calama (n = 1). Detailed demographic and genotyping information is listed in **S1 Table**. Analysis of drug resistance profiles showed that most isolates were pan-susceptible (n = 402) while some were resistant to any drug (n = 44) or multidrug resistant (n = 6). For a few cases, data regarding resistance was unavailable (n = 6). Three of the six MDR-TB strains belonged to SIT53/T1 and two were from Santiago. Among strains that were resistant to any antibiotic, our results showed that: (i) of the six belonging to SIT33, five were resistant to streptomycin; (ii) the six strains of SIT42 presented with a heterogeneous resistance, one or a mixture of antibiotics, and (iii) three out of a total of six strains of SIT4015 presented with some form of resistance throughout the study.

Spoligotyping of the 458 isolates revealed a total of 169 different patterns: 119 patterns (408 strains, 89.08% of sample population) corresponded to the shared-types or SITs when compared to the SITVIT2 database, and 50 patterns (50 strains, 10.92% of sample population) corresponded to orphan strains that have not yet been reported in literature. A total of 95/119 SITs (n = 357 isolates) matched a preexisting shared-type recorded in the database, whereas 24/119 SITs (n = 51 isolates) were newly created when they matched orphan strains recorded in the database (SIT4014 –SIT4037). The 50 orphan remaining strains were also recorded in the database. A detailed description of the 119 SITs (n = 408 isolates) and their corresponding spoligotyping defined lineages/sublineages are shown in **Table 1**.

**Table 1:** Description of 119 shared-types (SITs; n = 408 isolates) and corresponding spoligotyping defined lineages/sublineages starting from a total of 458 *M*. *tuberculosis* strains isolated in various cities in Chile.

SIT*	Spoligotype Description	Octal Number	Nb in study (%)	% in study vs. database	Lineage**	Unique vs. Clustered SIT***
**1**	⬜⬜⬜⬜⬜⬜⬜⬜⬜⬜⬜⬜⬜⬜⬜⬜⬜⬜⬜⬜⬜⬜⬜⬜⬜⬜⬜⬜⬜⬜⬜⬜⬜⬜⬛⬛⬛⬛⬛⬛⬛⬛⬛	000000000003771	2 (0.44)	0.02	Beijing	Clustered
**2**	⬜⬜⬜⬜⬜⬜⬜⬜⬜⬜⬜⬜⬜⬜⬜⬜⬜⬜⬜⬜⬜⬜⬜⬜⬛⬜⬜⬜⬜⬜⬜⬛⬜⬜⬜⬜⬛⬛⬛⬛⬛⬛⬛	000000004020771	1 (0.22)	0.21	H2	Unique
**4**	⬜⬜⬜⬜⬜⬜⬜⬜⬜⬜⬜⬜⬜⬜⬜⬜⬜⬜⬜⬜⬜⬜⬜⬜⬛⬛⬛⬛⬛⬛⬛⬛⬜⬜⬜⬜⬛⬛⬛⬛⬛⬛⬛	000000007760771	1 (0.22)	0.26	Unknown	Unique
**20**	⬛⬛⬜⬛⬛⬛⬛⬛⬛⬛⬛⬛⬛⬛⬛⬛⬛⬛⬛⬛⬜⬜⬜⬜⬛⬛⬛⬛⬛⬛⬛⬛⬜⬜⬜⬜⬛⬛⬛⬛⬛⬛⬛	677777607760771	3 (0.66)	0.33	LAM1	Clustered
**33**	⬛⬛⬛⬛⬛⬛⬛⬛⬜⬜⬜⬛⬛⬛⬛⬛⬛⬛⬛⬛⬜⬜⬜⬜⬛⬛⬛⬛⬛⬛⬛⬛⬜⬜⬜⬜⬛⬛⬛⬛⬛⬛⬛	776177607760771	44 (9.61)	3.25	LAM3	Clustered
**37**	⬛⬛⬛⬛⬛⬛⬛⬛⬛⬛⬛⬛⬜⬛⬛⬛⬛⬛⬛⬛⬛⬛⬛⬛⬛⬛⬛⬛⬛⬛⬛⬛⬜⬜⬜⬜⬛⬛⬛⬛⬛⬛⬛	777737777760771	23 (5.02)	4.23	T3	Clustered
**39**	⬛⬛⬛⬛⬛⬛⬛⬛⬛⬛⬛⬛⬛⬛⬛⬛⬛⬛⬜⬛⬛⬛⬜⬜⬛⬛⬛⬛⬛⬛⬛⬛⬜⬜⬜⬜⬛⬜⬜⬛⬛⬛⬛	777777347760471	1 (0.22)	0.67	T4-CEU1	Unique
**42**	⬛⬛⬛⬛⬛⬛⬛⬛⬛⬛⬛⬛⬛⬛⬛⬛⬛⬛⬛⬛⬜⬜⬜⬜⬛⬛⬛⬛⬛⬛⬛⬛⬜⬜⬜⬜⬛⬛⬛⬛⬛⬛⬛	777777607760771	43 (9.39)	1.21	LAM9	Clustered
**44**	⬛⬛⬛⬛⬛⬛⬛⬛⬛⬛⬛⬛⬛⬛⬛⬛⬛⬛⬛⬛⬛⬛⬜⬛⬛⬛⬛⬛⬛⬛⬛⬛⬜⬜⬜⬜⬛⬛⬛⬛⬛⬛⬛	777777757760771	1 (0.22)	0.46	T5	Unique
**47**	⬛⬛⬛⬛⬛⬛⬛⬛⬛⬛⬛⬛⬛⬛⬛⬛⬛⬛⬛⬛⬛⬛⬛⬛⬛⬜⬜⬜⬜⬜⬜⬛⬜⬜⬜⬜⬛⬛⬛⬛⬛⬛⬛	777777774020771	5 (1.09)	0.3	H1	Clustered
**50**	⬛⬛⬛⬛⬛⬛⬛⬛⬛⬛⬛⬛⬛⬛⬛⬛⬛⬛⬛⬛⬛⬛⬛⬛⬛⬛⬛⬛⬛⬛⬜⬛⬜⬜⬜⬜⬛⬛⬛⬛⬛⬛⬛	777777777720771	27 (5.9)	0.68	H3	Clustered
**51**	⬛⬛⬛⬛⬛⬛⬛⬛⬛⬛⬛⬛⬛⬛⬛⬛⬛⬛⬛⬛⬛⬛⬛⬛⬛⬛⬛⬛⬛⬛⬛⬛⬜⬜⬜⬜⬛⬛⬛⬜⬜⬜⬜	777777777760700	3 (0.66)	0.95	T1	Clustered
**52**	⬛⬛⬛⬛⬛⬛⬛⬛⬛⬛⬛⬛⬛⬛⬛⬛⬛⬛⬛⬛⬛⬛⬛⬛⬛⬛⬛⬛⬛⬛⬛⬛⬜⬜⬜⬜⬛⬛⬛⬜⬛⬛⬛	777777777760731	2 (0.44)	0.21	T2	Clustered
**53**	⬛⬛⬛⬛⬛⬛⬛⬛⬛⬛⬛⬛⬛⬛⬛⬛⬛⬛⬛⬛⬛⬛⬛⬛⬛⬛⬛⬛⬛⬛⬛⬛⬜⬜⬜⬜⬛⬛⬛⬛⬛⬛⬛	777777777760771	53 (11.57)	0.8	T1	Clustered
**58**	⬛⬛⬛⬛⬛⬛⬛⬛⬛⬛⬛⬛⬛⬛⬛⬛⬛⬛⬛⬜⬛⬛⬜⬛⬛⬛⬛⬛⬛⬛⬛⬛⬜⬜⬜⬜⬛⬛⬛⬛⬛⬛⬛	777777557760771	4 (0.87)	2.05	T5-Madrid2	Clustered
**60**	⬛⬛⬛⬛⬛⬛⬛⬛⬛⬛⬛⬛⬛⬛⬛⬛⬛⬛⬛⬛⬜⬜⬜⬜⬛⬛⬛⬛⬛⬛⬛⬛⬜⬜⬜⬜⬛⬛⬛⬜⬛⬛⬛	777777607760731	5 (1.09)	1.12	LAM4	Clustered
**61**	⬛⬛⬛⬛⬛⬛⬛⬛⬛⬛⬛⬛⬛⬛⬛⬛⬛⬛⬛⬛⬛⬛⬜⬜⬜⬛⬛⬛⬛⬛⬛⬛⬜⬜⬜⬜⬛⬛⬛⬛⬛⬛⬛	777777743760771	1 (0.22)	0.1	Cameroon	Unique
**62**	⬛⬛⬛⬛⬛⬛⬛⬛⬛⬛⬛⬛⬛⬛⬛⬛⬛⬛⬛⬛⬛⬛⬛⬛⬛⬜⬜⬜⬜⬜⬜⬛⬜⬜⬜⬜⬛⬛⬛⬜⬛⬛⬛	777777774020731	1 (0.22)	0.17	H1	Unique
**64**	⬛⬛⬛⬛⬛⬛⬛⬛⬛⬛⬛⬛⬛⬛⬛⬛⬛⬛⬛⬛⬜⬜⬜⬜⬛⬛⬛⬛⬜⬛⬛⬛⬜⬜⬜⬜⬛⬛⬛⬛⬛⬛⬛	777777607560771	8 (1.75)	1.94	LAM6	Clustered
**78**	⬛⬛⬛⬛⬛⬛⬛⬛⬛⬛⬛⬛⬛⬛⬛⬛⬛⬛⬛⬛⬛⬛⬛⬛⬛⬛⬛⬛⬛⬛⬛⬛⬜⬜⬜⬜⬛⬛⬛⬜⬜⬛⬛	777777777760711	1 (0.22)	1.45	T	Unique
**81**	⬛⬛⬛⬛⬛⬛⬛⬛⬛⬜⬛⬛⬛⬛⬛⬛⬛⬛⬛⬛⬜⬜⬜⬜⬛⬛⬛⬛⬛⬛⬛⬛⬜⬜⬜⬜⬛⬛⬛⬛⬛⬛⬛	777377607760771	1 (0.22)	2.63	LAM9	Unique
**91**	⬛⬛⬛⬜⬜⬜⬜⬜⬜⬜⬜⬜⬜⬛⬛⬛⬛⬜⬛⬛⬛⬛⬛⬛⬛⬛⬛⬛⬛⬛⬛⬛⬜⬜⬜⬜⬛⬛⬛⬛⬛⬛⬛	700036777760771	5 (1.09)	1.36	X3	Clustered
**93**	⬛⬛⬛⬛⬛⬛⬛⬛⬛⬛⬛⬛⬜⬛⬛⬛⬛⬛⬛⬛⬜⬜⬜⬜⬛⬛⬛⬛⬛⬛⬛⬛⬜⬜⬜⬜⬛⬛⬛⬛⬛⬛⬛	777737607760771	1 (0.22)	0.22	LAM5	Unique
**99**	⬛⬛⬛⬛⬜⬛⬛⬛⬛⬛⬛⬛⬛⬛⬛⬛⬛⬛⬛⬛⬛⬛⬛⬛⬛⬛⬛⬛⬛⬛⬜⬛⬜⬜⬜⬜⬛⬛⬛⬛⬛⬛⬛	757777777720771	2 (0.44)	2.41	H3	Clustered
**111**	⬛⬛⬛⬛⬛⬛⬛⬛⬜⬜⬜⬛⬛⬛⬜⬛⬛⬛⬛⬛⬜⬜⬜⬜⬛⬛⬛⬛⬛⬛⬛⬛⬜⬜⬜⬜⬛⬛⬛⬛⬛⬛⬛	776167607760771	1 (0.22)	3.13	LAM3	Unique
**118**	⬛⬛⬛⬛⬛⬛⬛⬛⬛⬛⬛⬛⬛⬛⬜⬛⬛⬛⬛⬛⬛⬛⬛⬛⬛⬛⬛⬛⬛⬛⬛⬛⬜⬜⬜⬜⬛⬛⬛⬛⬛⬛⬛	777767777760771	1 (0.22)	0.6	T1	Unique
**119**	⬛⬛⬛⬛⬛⬛⬛⬛⬛⬛⬛⬛⬛⬛⬛⬛⬛⬜⬛⬛⬛⬛⬛⬛⬛⬛⬛⬛⬛⬛⬛⬛⬜⬜⬜⬜⬛⬛⬛⬛⬛⬛⬛	777776777760771	1 (0.22)	0.09	X1	Unique
**124**	⬛⬛⬛⬛⬛⬛⬛⬛⬛⬛⬛⬛⬛⬛⬛⬛⬛⬛⬛⬛⬛⬛⬛⬛⬛⬛⬛⬛⬛⬛⬜⬜⬜⬜⬜⬜⬛⬛⬛⬛⬛⬛⬛	777777777700771	1 (0.22)	1.47	Unknown	Unique
**130**	⬛⬛⬛⬛⬛⬛⬛⬛⬜⬜⬜⬛⬛⬛⬛⬛⬛⬛⬛⬛⬜⬜⬜⬜⬛⬛⬛⬛⬛⬛⬛⬛⬜⬜⬜⬜⬛⬛⬛⬜⬛⬛⬛	776177607760731	2 (0.44)	1.55	LAM3	Clustered
**137**	⬛⬛⬛⬛⬛⬛⬛⬛⬛⬛⬛⬛⬛⬛⬛⬛⬛⬜⬛⬛⬛⬛⬛⬛⬛⬛⬛⬛⬛⬛⬛⬛⬜⬜⬜⬜⬛⬛⬜⬜⬜⬜⬛	777776777760601	3 (0.66)	0.29	X2	Clustered
**154**	⬛⬛⬛⬛⬜⬛⬛⬛⬛⬛⬛⬛⬛⬛⬛⬛⬛⬛⬛⬛⬛⬛⬛⬛⬛⬛⬛⬛⬛⬛⬛⬛⬜⬜⬜⬜⬛⬛⬛⬛⬛⬛⬛	757777777760771	1 (0.22)	0.88	T1	Unique
**162**	⬛⬛⬛⬛⬛⬛⬛⬛⬛⬛⬛⬛⬛⬛⬛⬛⬛⬛⬛⬛⬜⬜⬜⬜⬛⬛⬛⬛⬛⬛⬛⬛⬜⬜⬜⬜⬛⬛⬜⬛⬛⬛⬛	777777607760671	1 (0.22)	2.86	LAM9	Unique
**163**	⬛⬛⬛⬛⬛⬛⬛⬛⬛⬛⬛⬛⬛⬛⬛⬛⬛⬛⬛⬛⬜⬜⬜⬜⬛⬛⬛⬛⬛⬛⬛⬛⬜⬜⬜⬜⬛⬛⬛⬜⬜⬜⬜	777777607760700	1 (0.22)	2.5	LAM4	Unique
**180**	⬛⬛⬜⬛⬛⬛⬛⬛⬛⬛⬛⬛⬛⬛⬛⬛⬛⬛⬛⬛⬛⬛⬛⬛⬛⬛⬛⬛⬛⬛⬜⬛⬜⬜⬜⬜⬛⬛⬛⬛⬛⬛⬛	677777777720771	2 (0.44)	3.23	H3	Clustered
**194**	⬛⬛⬜⬛⬛⬛⬛⬛⬛⬛⬛⬛⬜⬛⬛⬛⬛⬛⬛⬛⬜⬜⬜⬜⬛⬛⬛⬛⬛⬛⬛⬛⬜⬜⬜⬜⬛⬛⬛⬜⬛⬛⬛	677737607760731	2 (0.44)	6.06	LAM2	Clustered
**195**	⬛⬛⬜⬛⬛⬛⬛⬛⬛⬛⬛⬛⬛⬛⬛⬛⬛⬛⬛⬛⬜⬜⬜⬜⬛⬛⬛⬛⬛⬜⬛⬛⬜⬜⬜⬜⬛⬛⬛⬛⬛⬛⬛	677777607660771	1 (0.22)	6.25	LAM1	Unique
**211**	⬛⬛⬛⬛⬛⬛⬛⬛⬜⬜⬜⬛⬜⬛⬛⬛⬛⬛⬛⬛⬜⬜⬜⬜⬛⬛⬛⬛⬛⬛⬛⬛⬜⬜⬜⬜⬛⬛⬛⬛⬛⬛⬛	776137607760771	12 (2.62)	10.17	LAM3	Clustered
**222**	⬛⬛⬛⬛⬛⬛⬛⬛⬛⬛⬛⬛⬛⬛⬛⬛⬜⬜⬜⬜⬜⬛⬛⬛⬛⬛⬛⬛⬜⬛⬛⬛⬜⬜⬜⬜⬛⬛⬛⬛⬛⬛⬛	777774077560771	4 (0.87)	3.96	Unknown	Clustered
**238**	⬛⬛⬛⬛⬛⬛⬛⬛⬛⬛⬛⬛⬛⬛⬛⬛⬛⬛⬛⬛⬛⬛⬛⬛⬛⬛⬛⬛⬛⬛⬜⬜⬜⬜⬜⬜⬜⬜⬛⬛⬛⬛⬛	777777777700171	1 (0.22)	5.56	Unknown	Unique
**239**	⬛⬛⬛⬛⬛⬛⬛⬛⬛⬛⬛⬛⬛⬛⬛⬛⬛⬛⬛⬛⬛⬛⬛⬛⬛⬛⬛⬛⬛⬛⬛⬛⬜⬜⬜⬜⬜⬜⬜⬜⬛⬛⬛	777777777760031	1 (0.22)	1.64	T2	Unique
**241**	⬛⬛⬛⬛⬛⬛⬛⬛⬛⬛⬛⬛⬛⬛⬛⬛⬛⬛⬛⬛⬛⬛⬛⬛⬛⬛⬛⬛⬛⬛⬛⬛⬜⬜⬜⬜⬛⬜⬜⬜⬜⬛⬛	777777777760411	1 (0.22)	3.57	T1	Unique
**244**	⬛⬛⬛⬛⬛⬛⬛⬛⬛⬛⬛⬛⬛⬛⬛⬛⬛⬛⬛⬛⬛⬛⬛⬛⬛⬛⬛⬛⬛⬛⬛⬛⬜⬜⬜⬜⬛⬛⬜⬜⬜⬜⬛	777777777760601	1 (0.22)	0.84	T1	Unique
**245**	⬛⬛⬛⬛⬛⬛⬛⬛⬛⬛⬛⬛⬛⬛⬛⬛⬛⬛⬛⬛⬛⬛⬛⬛⬛⬛⬛⬛⬛⬛⬛⬛⬜⬜⬜⬜⬛⬛⬜⬛⬛⬛⬛	777777777760671	1 (0.22)	4.76	T1	Unique
**283**	⬛⬛⬛⬛⬛⬛⬛⬛⬛⬛⬛⬛⬛⬛⬛⬛⬛⬛⬛⬛⬛⬜⬜⬜⬛⬜⬜⬜⬜⬜⬜⬛⬜⬜⬜⬜⬛⬛⬛⬛⬛⬛⬛	777777704020771	6 (1.31)	8.11	H1	Clustered
**393**	⬛⬛⬛⬛⬛⬛⬛⬛⬛⬛⬛⬛⬛⬜⬛⬛⬛⬛⬛⬛⬛⬛⬛⬛⬛⬛⬛⬛⬛⬛⬛⬛⬜⬜⬜⬜⬛⬛⬛⬛⬛⬛⬛	777757777760771	1 (0.22)	2.44	T1	Unique
**430**	⬛⬛⬛⬜⬜⬜⬛⬜⬜⬜⬜⬜⬜⬜⬜⬜⬛⬛⬜⬛⬛⬛⬜⬜⬛⬛⬛⬛⬛⬛⬛⬛⬜⬜⬜⬜⬛⬜⬜⬛⬛⬛⬛	704003347760471	1 (0.22)	2.7	T4-CEU1	Unique
**434**	⬛⬛⬛⬛⬛⬜⬜⬜⬜⬛⬛⬛⬛⬛⬛⬛⬛⬛⬛⬛⬛⬛⬛⬛⬛⬛⬛⬛⬛⬛⬛⬜⬜⬜⬜⬜⬜⬛⬛⬛⬛⬛⬛	760777777740371	1 (0.22)	16.67	T1	Unique
**435**	⬛⬛⬛⬛⬛⬜⬜⬛⬛⬛⬛⬛⬛⬛⬛⬛⬛⬛⬛⬛⬜⬜⬜⬜⬛⬛⬛⬛⬛⬛⬛⬛⬜⬜⬜⬜⬛⬛⬛⬛⬛⬛⬛	763777607760771	1 (0.22)	16.67	LAM9	Unique
**450**	⬛⬛⬛⬛⬛⬛⬛⬛⬛⬛⬛⬛⬛⬛⬛⬛⬛⬜⬛⬛⬛⬛⬛⬛⬜⬜⬜⬜⬜⬜⬜⬜⬜⬜⬜⬜⬜⬜⬜⬜⬜⬜⬜	777776770000000	1 (0.22)	0.8	Unknown	Unique
**504**	⬛⬛⬛⬛⬛⬛⬛⬛⬛⬛⬛⬛⬜⬛⬛⬛⬛⬛⬛⬛⬛⬜⬛⬛⬛⬛⬛⬛⬛⬛⬛⬛⬜⬜⬜⬜⬛⬛⬛⬛⬛⬛⬛	777737737760771	1 (0.22)	4.35	T3	Unique
**720**	⬛⬛⬛⬛⬛⬛⬛⬛⬜⬜⬜⬛⬛⬛⬛⬛⬛⬛⬛⬛⬜⬜⬜⬜⬛⬛⬛⬛⬛⬛⬛⬛⬜⬜⬜⬜⬜⬛⬛⬛⬛⬛⬛	776177607760371	6 (1.31)	54.55	LAM3	Clustered
**753**	⬛⬜⬜⬛⬛⬛⬛⬛⬛⬛⬛⬛⬛⬛⬛⬛⬛⬛⬛⬛⬜⬜⬜⬜⬛⬛⬛⬛⬛⬛⬛⬛⬜⬜⬜⬜⬛⬛⬛⬛⬛⬛⬛	477777607760771	1 (0.22)	4.55	LAM1	Unique
**787**	⬛⬛⬛⬛⬛⬛⬛⬛⬛⬛⬛⬛⬛⬛⬛⬛⬛⬛⬛⬛⬛⬛⬛⬛⬛⬛⬛⬛⬛⬛⬛⬛⬜⬜⬜⬜⬜⬜⬜⬛⬛⬛⬛	777777777760071	1 (0.22)	12.5	T1	Unique
**798**	⬛⬜⬜⬜⬛⬛⬛⬛⬛⬛⬛⬛⬛⬛⬛⬛⬛⬛⬛⬛⬛⬛⬛⬛⬛⬛⬛⬛⬛⬛⬛⬛⬜⬜⬜⬜⬛⬛⬛⬛⬛⬛⬛	437777777760771	2 (0.44)	33.33	T1	Clustered
**880**	⬛⬛⬛⬛⬛⬛⬛⬛⬛⬛⬛⬛⬛⬛⬛⬛⬛⬛⬜⬜⬜⬜⬜⬜⬜⬜⬜⬛⬛⬛⬛⬛⬜⬜⬜⬜⬛⬛⬛⬛⬛⬛⬛	777777000760771	1 (0.22)	10	T1	Unique
**900**	⬛⬛⬜⬛⬛⬛⬛⬛⬛⬛⬛⬛⬛⬛⬛⬛⬛⬛⬛⬛⬜⬜⬜⬜⬛⬛⬛⬛⬛⬛⬛⬜⬜⬜⬜⬜⬜⬜⬜⬜⬜⬛⬛	677777607740011	1 (0.22)	25	LAM	Unique
**914**	⬛⬛⬛⬛⬛⬛⬛⬛⬜⬜⬛⬛⬛⬛⬛⬛⬛⬛⬛⬛⬛⬛⬛⬛⬛⬛⬛⬛⬛⬛⬜⬛⬜⬜⬜⬜⬛⬛⬛⬛⬛⬛⬛	776377777720771	1 (0.22)	1.45	Unknown	Unique
**948**	⬛⬛⬛⬛⬛⬛⬛⬛⬛⬛⬛⬛⬛⬛⬛⬛⬛⬛⬛⬛⬛⬛⬛⬜⬜⬜⬜⬜⬜⬜⬜⬛⬜⬜⬜⬜⬛⬛⬜⬜⬜⬛⬛	777777760020611	2 (0.44)	14.29	H3	Clustered
**1073**	⬛⬛⬛⬛⬛⬛⬛⬛⬛⬛⬛⬜⬜⬛⬛⬛⬛⬛⬛⬛⬛⬛⬛⬛⬛⬛⬛⬛⬛⬛⬛⬛⬜⬜⬜⬜⬛⬛⬛⬛⬛⬛⬛	777637777760771	1 (0.22)	12.5	T1	Unique
**1105**	⬛⬛⬛⬛⬛⬛⬛⬛⬛⬛⬛⬛⬛⬛⬛⬜⬛⬛⬛⬛⬛⬛⬛⬛⬛⬛⬛⬛⬛⬛⬛⬛⬜⬜⬜⬜⬛⬛⬛⬛⬛⬛⬛	777773777760771	1 (0.22)	2.78	T1	Unique
**1116**	⬛⬛⬜⬜⬜⬜⬛⬛⬛⬛⬛⬛⬛⬛⬛⬛⬛⬛⬛⬛⬛⬛⬛⬛⬛⬛⬛⬛⬛⬛⬜⬛⬜⬜⬜⬜⬛⬛⬛⬛⬛⬛⬛	607777777720771	1 (0.22)	33.33	H3	Unique
**1220**	⬛⬛⬛⬛⬜⬛⬛⬜⬜⬜⬛⬛⬛⬛⬛⬛⬛⬛⬛⬛⬜⬜⬜⬜⬛⬛⬛⬛⬛⬛⬛⬛⬜⬜⬜⬜⬛⬛⬛⬜⬜⬜⬜	754377607760700	1 (0.22)	16.67	LAM	Unique
**1232**	⬛⬛⬛⬛⬛⬛⬛⬛⬛⬛⬛⬛⬛⬛⬛⬛⬜⬜⬜⬜⬜⬛⬛⬛⬛⬛⬛⬛⬜⬛⬛⬛⬜⬜⬜⬜⬛⬛⬛⬜⬛⬛⬛	777774077560731	1 (0.22)	10	T2	Unique
**1259**	⬛⬛⬛⬛⬛⬛⬛⬛⬛⬛⬛⬛⬛⬜⬜⬜⬜⬜⬜⬜⬜⬜⬜⬜⬜⬜⬜⬜⬜⬛⬛⬛⬜⬜⬜⬜⬛⬛⬛⬛⬛⬛⬛	777740000160771	1 (0.22)	25	T1	Unique
**1277**	⬛⬛⬛⬛⬛⬛⬛⬛⬛⬛⬛⬛⬛⬛⬛⬛⬛⬛⬜⬛⬜⬜⬜⬜⬛⬛⬛⬛⬛⬛⬛⬛⬜⬜⬜⬜⬛⬛⬛⬛⬛⬛⬛	777777207760771	6 (1.31)	16.67	LAM9	Clustered
**1476**	⬛⬛⬛⬜⬛⬛⬛⬛⬜⬜⬜⬛⬛⬛⬛⬛⬛⬛⬛⬛⬜⬜⬜⬜⬛⬛⬛⬛⬛⬛⬜⬜⬜⬜⬜⬜⬜⬜⬛⬛⬛⬛⬛	736177607700171	1 (0.22)	3.13	AFRI_2	Unique
**1560**	⬛⬛⬛⬜⬜⬜⬜⬛⬛⬛⬛⬛⬛⬛⬛⬛⬛⬛⬛⬛⬛⬛⬛⬛⬛⬛⬛⬛⬛⬛⬛⬛⬜⬜⬜⬜⬛⬛⬛⬛⬛⬛⬛	703777777760771	2 (0.44)	22.22	T1	Clustered
**1626**	⬛⬛⬛⬛⬛⬛⬛⬛⬛⬛⬛⬛⬛⬛⬛⬛⬛⬛⬛⬛⬛⬛⬛⬛⬛⬛⬜⬛⬛⬛⬛⬛⬜⬜⬜⬜⬛⬛⬛⬛⬛⬛⬛	777777776760771	1 (0.22)	7.69	T1	Unique
**1685**	⬛⬛⬛⬛⬛⬛⬛⬛⬜⬜⬜⬜⬛⬛⬛⬛⬛⬛⬛⬛⬜⬜⬜⬜⬛⬛⬛⬛⬛⬛⬛⬛⬜⬜⬜⬜⬛⬛⬛⬛⬛⬛⬛	776077607760771	3 (0.66)	37.5	LAM3	Clustered
**1706**	⬛⬛⬛⬜⬜⬛⬛⬛⬛⬛⬛⬛⬛⬛⬛⬛⬛⬛⬛⬛⬜⬜⬜⬜⬛⬛⬛⬛⬛⬛⬛⬛⬜⬜⬜⬜⬛⬛⬛⬛⬛⬛⬛	717777607760771	1 (0.22)	10	LAM9	Unique
**1710**	⬛⬛⬛⬛⬛⬛⬛⬛⬛⬛⬛⬛⬜⬛⬛⬛⬛⬛⬛⬛⬜⬜⬜⬜⬛⬛⬛⬛⬛⬛⬛⬛⬜⬜⬜⬜⬛⬛⬛⬜⬛⬛⬛	777737607760731	4 (0.87)	36.36	LAM	Clustered
**1841**	⬛⬛⬛⬛⬛⬛⬛⬛⬜⬜⬜⬛⬛⬛⬛⬛⬛⬛⬛⬛⬜⬜⬜⬜⬛⬛⬛⬛⬛⬛⬛⬜⬜⬜⬜⬜⬛⬛⬛⬛⬛⬛⬛	776177607740771	1 (0.22)	16.67	LAM3	Unique
**1890**	⬛⬛⬛⬛⬛⬛⬛⬛⬛⬛⬛⬛⬛⬛⬛⬛⬛⬛⬛⬛⬛⬜⬜⬜⬛⬛⬛⬛⬛⬛⬛⬛⬜⬜⬜⬜⬛⬛⬛⬜⬛⬛⬛	777777707760731	1 (0.22)	8.33	T2	Unique
**2014**	⬛⬛⬛⬛⬛⬛⬛⬛⬜⬜⬜⬛⬛⬛⬛⬛⬛⬛⬛⬛⬜⬜⬜⬜⬛⬛⬛⬜⬛⬛⬛⬛⬜⬜⬜⬜⬛⬛⬛⬛⬛⬛⬛	776177607360771	1 (0.22)	20	LAM3	Unique
**2015**	⬛⬛⬛⬜⬛⬛⬛⬛⬜⬜⬜⬛⬛⬛⬛⬛⬛⬛⬛⬛⬜⬜⬜⬜⬛⬛⬛⬛⬛⬛⬛⬛⬜⬜⬜⬜⬛⬛⬛⬛⬛⬛⬛	736177607760771	2 (0.44)	33.33	LAM3	Clustered
**2028**	⬛⬛⬛⬛⬛⬛⬛⬛⬛⬛⬛⬛⬛⬛⬛⬛⬛⬛⬛⬛⬜⬜⬜⬜⬛⬜⬜⬜⬜⬜⬜⬛⬜⬜⬜⬜⬛⬛⬛⬛⬛⬛⬛	777777604020771	1 (0.22)	10	Unknown	Unique
**2045**	⬛⬛⬛⬛⬛⬛⬛⬛⬜⬜⬜⬜⬜⬛⬛⬛⬛⬛⬛⬛⬜⬜⬜⬜⬛⬛⬛⬛⬛⬛⬛⬛⬜⬜⬜⬜⬛⬛⬛⬛⬛⬛⬛	776037607760771	1 (0.22)	6.25	LAM3	Unique
**2054**	⬛⬛⬜⬛⬛⬛⬛⬛⬛⬛⬛⬛⬛⬛⬛⬛⬛⬛⬛⬛⬜⬜⬜⬜⬛⬛⬛⬛⬛⬛⬛⬛⬜⬜⬜⬜⬜⬛⬛⬛⬛⬛⬛	677777607760371	1 (0.22)	8.33	LAM	Unique
**2070**	⬛⬛⬛⬛⬛⬛⬛⬛⬛⬛⬛⬛⬛⬛⬛⬛⬛⬛⬛⬛⬜⬜⬛⬛⬛⬛⬛⬛⬛⬛⬛⬛⬜⬜⬜⬜⬛⬛⬛⬛⬛⬛⬛	777777637760771	4 (0.87)	23.53	T1	Clustered
**2353**	⬛⬛⬛⬛⬛⬛⬛⬛⬜⬜⬜⬛⬛⬛⬛⬛⬛⬛⬛⬛⬜⬜⬜⬜⬜⬜⬜⬜⬜⬜⬜⬜⬜⬜⬜⬜⬜⬜⬜⬜⬜⬜⬜	776177600000000	2 (0.44)	18.18	Unknown	Clustered
**2366**	⬛⬛⬛⬛⬛⬛⬛⬛⬛⬛⬛⬛⬛⬛⬛⬛⬛⬛⬛⬛⬛⬛⬛⬛⬛⬛⬛⬛⬛⬛⬛⬛⬜⬜⬜⬜⬜⬜⬛⬛⬛⬛⬛	777777777760171	1 (0.22)	16.67	T1	Unique
**2402**	⬛⬛⬛⬛⬛⬛⬛⬛⬜⬜⬜⬛⬛⬛⬛⬛⬛⬛⬛⬛⬜⬜⬜⬜⬛⬛⬛⬛⬛⬛⬛⬛⬜⬜⬜⬜⬛⬛⬛⬜⬜⬛⬛	776177607760711	1 (0.22)	25	LAM3	Unique
**2512**	⬛⬛⬜⬛⬜⬛⬛⬛⬛⬛⬛⬛⬛⬜⬛⬛⬛⬛⬛⬛⬜⬜⬜⬜⬛⬛⬛⬛⬛⬛⬛⬛⬜⬜⬜⬜⬛⬛⬛⬛⬛⬛⬛	657757607760771	1 (0.22)	16.67	LAM1	Unique
**2745**	⬛⬜⬛⬛⬛⬛⬛⬛⬛⬛⬛⬛⬛⬛⬛⬛⬛⬛⬛⬛⬜⬜⬜⬜⬛⬛⬛⬛⬛⬛⬛⬛⬜⬜⬜⬜⬛⬛⬛⬛⬜⬛⬛	577777607760751	2 (0.44)	40	LAM9	Clustered
**2885**	⬛⬛⬛⬛⬛⬛⬛⬛⬛⬛⬛⬛⬛⬛⬛⬛⬛⬛⬛⬜⬜⬛⬛⬛⬛⬛⬛⬛⬛⬛⬜⬛⬜⬜⬜⬜⬛⬛⬛⬛⬛⬛⬛	777777477720771	1 (0.22)	6.25	H3	Unique
**2911**	⬛⬛⬛⬛⬛⬛⬛⬛⬛⬛⬛⬛⬜⬛⬛⬛⬛⬛⬛⬛⬛⬛⬛⬛⬛⬛⬛⬛⬛⬛⬜⬜⬜⬜⬜⬜⬛⬛⬛⬛⬛⬛⬛	777737777700771	1 (0.22)	33.33	Unknown	Unique
**2941**	⬜⬜⬜⬛⬛⬛⬛⬜⬜⬜⬛⬛⬛⬛⬛⬛⬛⬛⬛⬛⬜⬜⬜⬜⬛⬛⬛⬛⬛⬛⬛⬛⬜⬜⬜⬜⬛⬛⬛⬜⬜⬜⬜	074377607760700	1 (0.22)	25	LAM	Unique
**3056**	⬛⬛⬛⬛⬛⬛⬛⬛⬛⬛⬛⬛⬛⬛⬛⬛⬛⬛⬛⬛⬛⬛⬛⬛⬛⬛⬛⬛⬜⬛⬛⬛⬜⬜⬜⬜⬛⬛⬛⬜⬛⬛⬛	777777777560731	1 (0.22)	16.67	T2	Unique
**3127**	⬜⬜⬜⬜⬜⬜⬜⬜⬜⬜⬜⬜⬜⬜⬜⬜⬜⬜⬜⬜⬜⬜⬜⬛⬛⬛⬛⬛⬛⬛⬛⬛⬜⬜⬜⬜⬛⬛⬛⬛⬛⬛⬛	000000017760771	1 (0.22)	20	Unknown	Unique
**3251**	⬛⬛⬛⬛⬛⬛⬛⬛⬜⬜⬜⬛⬛⬛⬛⬛⬛⬛⬛⬛⬛⬛⬛⬛⬛⬛⬛⬛⬛⬛⬜⬛⬜⬜⬜⬜⬛⬛⬛⬛⬛⬛⬛	776177777720771	1 (0.22)	25	H3	Unique
**3472**	⬛⬛⬛⬛⬛⬛⬛⬛⬜⬜⬜⬛⬛⬛⬛⬛⬛⬛⬛⬛⬜⬜⬜⬜⬛⬛⬛⬛⬛⬛⬛⬛⬜⬜⬜⬜⬛⬛⬛⬛⬛⬛⬜	776177607760770	1 (0.22)	33.33	LAM3	Unique
**3491**	⬛⬛⬛⬛⬛⬛⬛⬛⬜⬜⬜⬛⬛⬛⬛⬛⬛⬛⬛⬛⬜⬜⬜⬜⬛⬛⬛⬛⬛⬛⬛⬛⬜⬜⬜⬜⬛⬛⬜⬛⬛⬛⬛	776177607760671	1 (0.22)	25	LAM3	Unique
**3934**	⬛⬜⬜⬜⬛⬛⬛⬛⬛⬛⬛⬛⬛⬛⬛⬛⬛⬛⬛⬛⬛⬛⬛⬛⬛⬛⬛⬛⬛⬛⬛⬛⬜⬜⬜⬜⬛⬛⬛⬜⬛⬛⬛	437777777760731	1 (0.22)	20	T2	Unique
**3965**	⬛⬛⬛⬜⬜⬜⬛⬛⬛⬛⬛⬛⬛⬛⬛⬛⬜⬜⬜⬜⬜⬛⬛⬛⬛⬛⬛⬛⬜⬛⬛⬛⬜⬜⬜⬜⬛⬛⬛⬛⬛⬛⬛	707774077560771	1 (0.22)	7.69	T1	Unique
**3967**	⬛⬛⬛⬛⬜⬜⬜⬜⬛⬛⬛⬛⬛⬛⬛⬛⬜⬜⬜⬜⬜⬛⬛⬛⬛⬛⬛⬛⬜⬛⬛⬛⬜⬜⬜⬜⬛⬛⬛⬛⬛⬛⬛	741774077560771	1 (0.22)	33.33	T1	Unique
4014*	⬛⬛⬛⬜⬜⬜⬜⬛⬛⬛⬛⬛⬛⬛⬛⬛⬛⬜⬛⬛⬛⬛⬛⬛⬛⬛⬛⬛⬛⬛⬛⬛⬜⬜⬜⬜⬛⬛⬛⬛⬛⬛⬛	703776777760771	7 (1.53)	100	X1	Clustered
4015*	⬛⬛⬛⬛⬛⬛⬛⬛⬜⬜⬜⬛⬛⬛⬛⬛⬛⬛⬛⬛⬛⬜⬜⬜⬜⬛⬛⬛⬛⬛⬛⬛⬛⬜⬜⬜⬜⬛⬛⬛⬛⬛⬛	776177703770371	7 (1.53)	100	Unknown	Clustered
4016*	⬛⬛⬛⬛⬛⬛⬛⬛⬛⬛⬛⬛⬛⬛⬛⬛⬛⬛⬛⬛⬛⬛⬛⬛⬛⬜⬜⬜⬜⬛⬜⬜⬜⬜⬜⬜⬛⬛⬛⬛⬛⬛⬛	777777774100771	2 (0.44)	100	Unknown	Clustered
4017*	⬛⬛⬛⬛⬛⬛⬛⬛⬛⬛⬛⬛⬜⬛⬛⬛⬛⬛⬛⬛⬜⬜⬛⬛⬛⬛⬛⬛⬛⬛⬛⬛⬜⬜⬜⬜⬛⬛⬛⬛⬛⬛⬛	777737637760771	3 (0.66)	75	T3	Clustered
4018*	⬛⬛⬛⬛⬛⬛⬛⬛⬛⬜⬛⬛⬛⬛⬛⬛⬛⬛⬛⬛⬜⬜⬛⬛⬛⬛⬜⬜⬜⬜⬜⬜⬜⬜⬜⬜⬛⬛⬛⬛⬛⬛⬛	777377636000771	2 (0.44)	100	Unknown	Clustered
4019*	⬛⬛⬛⬛⬛⬛⬛⬛⬜⬜⬜⬛⬛⬛⬛⬛⬛⬛⬛⬛⬜⬜⬜⬜⬛⬛⬛⬛⬛⬛⬜⬛⬜⬜⬜⬜⬛⬛⬛⬛⬛⬛⬛	776177607720771	1 (0.22)	50	H3	Unique
4020*	⬛⬛⬛⬛⬛⬛⬛⬛⬛⬜⬛⬛⬛⬜⬜⬛⬛⬛⬛⬛⬛⬛⬛⬛⬛⬛⬛⬛⬛⬛⬛⬛⬜⬜⬜⬜⬛⬛⬜⬛⬛⬛⬛	777347777760671	1 (0.22)	50	T1	Unique
4021*	⬛⬛⬛⬛⬛⬛⬛⬛⬛⬛⬛⬛⬜⬛⬛⬛⬛⬛⬛⬛⬛⬛⬛⬛⬛⬛⬛⬛⬛⬛⬜⬛⬜⬜⬜⬜⬛⬛⬜⬛⬛⬛⬛	777737777720671	1 (0.22)	50	H3	Unique
4022*	⬛⬛⬛⬛⬛⬛⬛⬛⬛⬛⬛⬛⬛⬛⬛⬛⬛⬛⬜⬛⬛⬛⬛⬜⬛⬜⬜⬜⬜⬜⬜⬛⬜⬜⬜⬜⬛⬛⬛⬛⬛⬛⬛	777777364020771	1 (0.22)	50	H1	Unique
4023*	⬛⬛⬛⬛⬛⬛⬛⬛⬛⬛⬛⬛⬛⬛⬛⬛⬜⬜⬜⬜⬜⬛⬛⬛⬛⬛⬛⬛⬜⬛⬛⬛⬜⬜⬜⬜⬛⬛⬛⬜⬜⬛⬛	777774077560711	1 (0.22)	50	T1	Unique
4024*	⬛⬛⬜⬛⬛⬜⬜⬛⬛⬛⬛⬛⬛⬛⬛⬛⬛⬛⬛⬛⬛⬛⬛⬛⬛⬛⬛⬛⬛⬛⬜⬛⬜⬜⬜⬜⬛⬛⬛⬛⬛⬛⬛	663777777720771	3 (0.66)	100	H3	Clustered
4025*	⬛⬛⬛⬛⬛⬛⬛⬛⬛⬛⬛⬛⬛⬛⬜⬜⬛⬛⬜⬛⬛⬛⬜⬜⬛⬛⬛⬛⬛⬛⬛⬛⬜⬜⬜⬜⬛⬜⬜⬛⬛⬛⬛	777763347760471	4 (0.87)	100	T4-CEU1	Clustered
4026*	⬛⬛⬛⬛⬛⬜⬜⬛⬛⬛⬛⬛⬛⬛⬛⬛⬛⬛⬛⬛⬜⬜⬜⬜⬛⬛⬛⬛⬛⬛⬛⬛⬜⬜⬜⬜⬛⬛⬛⬜⬜⬜⬜	763777607760700	2 (0.44)	100	LAM4	Clustered
4027*	⬛⬛⬛⬛⬛⬛⬜⬛⬜⬜⬜⬛⬛⬛⬛⬛⬛⬛⬛⬛⬜⬜⬜⬜⬛⬛⬛⬛⬛⬛⬛⬛⬜⬜⬜⬜⬛⬛⬛⬛⬛⬛⬛	772177607760771	1 (0.22)	50	LAM3	Unique
4028*	⬛⬛⬛⬜⬜⬜⬜⬜⬜⬜⬜⬜⬜⬛⬛⬛⬛⬜⬛⬛⬛⬛⬛⬛⬛⬜⬜⬜⬛⬛⬛⬛⬜⬜⬜⬜⬛⬛⬛⬛⬛⬛⬛	700036774360771	1 (0.22)	50	X3	Unique
4029*	⬛⬛⬛⬛⬛⬛⬛⬛⬛⬛⬛⬛⬛⬛⬛⬛⬛⬛⬛⬛⬛⬜⬜⬛⬛⬛⬛⬛⬜⬛⬛⬛⬜⬜⬜⬜⬛⬛⬛⬛⬛⬛⬛	777777717560771	1 (0.22)	50	T1	Unique
4030*	⬜⬜⬜⬜⬛⬛⬛⬛⬛⬛⬛⬛⬛⬛⬛⬛⬛⬛⬛⬛⬛⬜⬜⬜⬜⬜⬜⬜⬜⬜⬜⬜⬜⬜⬜⬜⬛⬛⬜⬜⬜⬛⬛	037777700000611	2 (0.44)	100	Unknown	Clustered
4031*	⬛⬛⬛⬛⬛⬛⬛⬛⬛⬛⬛⬛⬛⬛⬛⬛⬜⬜⬜⬜⬜⬜⬛⬛⬛⬛⬛⬛⬜⬛⬛⬛⬜⬜⬜⬜⬛⬛⬛⬛⬛⬛⬛	777774037560771	2 (0.44)	100	T1	Clustered
4032*	⬛⬛⬛⬛⬛⬛⬛⬛⬜⬜⬜⬜⬛⬛⬛⬛⬛⬛⬛⬛⬜⬜⬜⬜⬛⬛⬜⬜⬜⬛⬛⬛⬜⬜⬜⬜⬛⬛⬛⬛⬛⬛⬛	776077606160771	2 (0.44)	100	LAM3	Clustered
4033*	⬛⬛⬛⬛⬛⬛⬛⬛⬜⬜⬜⬛⬜⬛⬛⬛⬛⬛⬛⬛⬜⬜⬜⬜⬛⬛⬛⬛⬛⬛⬛⬛⬜⬜⬜⬜⬜⬛⬛⬛⬛⬛⬛	776137607760371	1 (0.22)	50	LAM3	Unique
4034*	⬛⬛⬛⬛⬛⬛⬛⬛⬜⬜⬜⬛⬛⬛⬛⬛⬛⬛⬛⬜⬜⬜⬜⬛⬛⬛⬛⬛⬛⬛⬛⬛⬜⬜⬜⬜⬛⬛⬛⬛⬛⬛⬛	776177417760771	2 (0.44)	100	T1	Clustered
4035*	⬛⬛⬜⬜⬜⬜⬜⬜⬛⬛⬛⬛⬛⬛⬛⬛⬛⬛⬛⬛⬛⬛⬛⬛⬛⬛⬛⬛⬛⬛⬛⬛⬜⬜⬜⬜⬛⬛⬛⬛⬛⬛⬛	601777777760771	2 (0.44)	100	T1	Clustered
4036*	⬛⬛⬛⬛⬛⬛⬛⬛⬛⬛⬛⬛⬛⬛⬛⬛⬛⬛⬛⬛⬜⬜⬜⬜⬛⬛⬛⬜⬜⬛⬛⬛⬜⬜⬜⬜⬛⬛⬛⬜⬛⬛⬛	777777607160731	1 (0.22)	50	LAM4	Unique
4037*	⬛⬛⬛⬛⬛⬛⬛⬛⬛⬛⬛⬛⬛⬛⬛⬛⬛⬛⬛⬛⬛⬜⬛⬛⬛⬜⬜⬜⬜⬜⬜⬛⬜⬜⬜⬜⬛⬛⬛⬛⬛⬛⬛	777777734020771	1 (0.22)	50	H1	Unique

* A total of 95/119 SITs containing 357 isolates matched a preexisting shared-type in the database, whereas 24/119 SITs (n = 51 isolates) were newly created. A total of 46/119 SITs containing 335 isolates were clustered within this study (2 to 53 isolates per cluster) while 73/119 SITs containing 73 strains were unique (for total unique strains, one should add to this number the 50 orphan strains, which brings the number of unclustered isolates in this study to 123/458 or 26.86%, and clustered isolates to 335/458 or 73.14%). Note that SIT followed by an asterisk indicates "newly created” SIT due to 2 or more strains belonging to an identical new pattern within this study or after a match with an orphan in the database; SIT designations followed by number of strains: 4014* this study n = 7; 4015* this study n = 7; 4016* this study n = 2; 4017* this study n = 3, VEN n = 1; 4018* this study n = 2; 4019* this study n = 1, ESP n = 1; 4020* this study n = 1, ARG n = 1; 4021* this study n = 1, IDN n = 1; 4022* this study n = 1, TUN n = 1; 4023* this study n = 1, PER n = 1; 4024* this study n = 3; 4025* this study n = 4; 4026* this study n = 2; 4027* this study n = 1, ESP n = 1; 4028* this study n = 1, PER n = 1; 4029* this study n = 1, BRA n = 1; 4030* this study n = 2; 4031* this study n = 2; 4032* this study n = 2; 4033* this study n = 1, ITA n = 1; 4034* this study n = 2; 4035* this study n = 2; 4036* this study n = 1, BRA n = 1; 4037* this study n = 1, BRA n = 1. The 3 letter country codes are according to http://en.wikipedia.org/wiki/ISO_3166-1_alpha-3.

** Lineage designations according to SITVIT2 using revised SpolDB4 rules; “Unknown” designates patterns with signatures that do not belong to any of the major lineages described in the database.

*** Clustered strains correspond to a similar spoligotype pattern shared by 2 or more strains “within this study”; as opposed to unique strains harboring a spoligotype pattern that does not match with another strain from this study. Unique strains matching a preexisting pattern in the SITVIT2 database are classified as SITs, whereas in case of no match, they are designated as “orphan”

**Fig 1:**
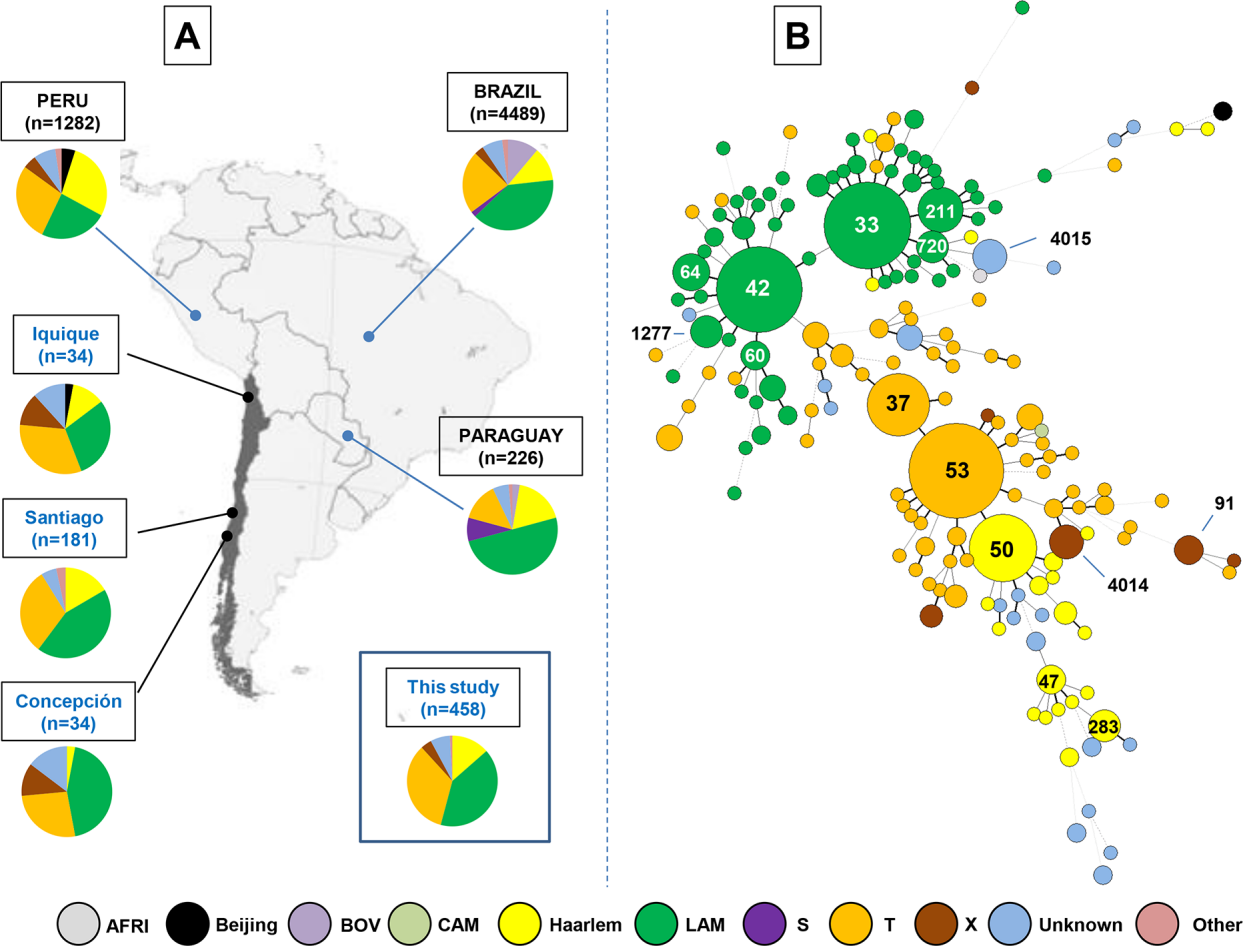
Phylogeographical distribution of *M*. *tuberculosis* lineages identified in our study and MST illustrating evolutionary relationships. (A) Phylogeographical distribution of major *M*. *tuberculosis* lineages in the 3 most important cities in our study (Santiago, Iquique, Concepción) as well as following neighboring countries: Peru, Brazil, and Paraguay (referring to the SITVIT2 database). (B) A minimum spanning tree (MST) illustrating evolutionary relationships between spoligotypes in various cities of Chile (n = 458 isolates). The phylogenetic tree connects each genotype based on degree of changes required to go from one allele to another. The structure of the tree is represented by branches (continuous vs. dashed and dotted lines) and circles representing each individual pattern. Note that the length of the branches represents the distance between patterns while the complexity of the lines (continuous, gray dashed and gray dotted) denotes the number of allele/spacer changes between two patterns: solid lines, 1 or 2 or 3 changes (thicker ones indicate a single change, while the thinner ones indicate 2 or 3 changes); gray dashed lines represent 4 changes; and gray dotted lines represent 5 or more changes. The size of the circle is proportional to the total number of isolates in our study, illustrating unique isolates (smaller nodes) versus clustered isolates (bigger nodes). The color of the circles indicates the phylogenetic lineage to which the specific pattern belongs. The labels of nodes indicate predominant SITs in study (containing at least 5 or more isolates).

For the 458 isolates analyzed in this study, distribution of lineages was as follow: LAM (n = 186 isolates, or 40.61%); T (n = 156 isolates, or 34.06%); Haarlem (n = 62 isolates, or 13.54%); X (n = 18 isolates, or 3.93%); Beijing (n = 2 isolates, or 0.44%); AFRI (n = 1 isolate, or 0.22%); Cameroon (n = 1 isolate, or 0.22%); and Unknown (n = 32 isolates, or 6.99%) (**S1 Table**). We observed significant differences in prevalence when the proportion of LAM, T, Haarlem and X lineages were compared within three of Chile’s most prominent cities, viz., Santiago, Concepción and Iquique (p-value = 0.0071). The observed proportion of Haarlem was remarkably lower in Concepción city (2.94%) as compared to Santiago (16.57%) and Iquique (11.76%). Regarding other lineages: (i) Beijing lineage was represented by about 3% of strains in Iquique and Santiago city; (ii) LAM lineage represented around 44% of strains in Santiago and Concepción, but only 29% in Iquique city; (iii) X lineage was more noticeable in Iquique and Concepción, representing around 12% of isolates, but in Santiago its incidence was less than 2%; (iv) the unknown strain was conspicuous in Iquique (11.8%) and Concepción (14.7%) but comparatively less prevalent in Santiago (6%) (**Fig 1A**).

A total of 257 isolates (56.11% of all 458) were clustered in fifteen of the most frequent spoligotypes (predominant SITs representing >1%, i.e., n = 5 or more isolates in our study): SIT53/T1 (n = 53; 11.57%), SIT33/LAM3 (n = 44; 9.61%), SIT42/LAM9 (n = 43; 9.39%), SIT50/H3 (n = 27; 5.90%), SIT37/T3 (n = 23; 5.02%), SIT211/LAM3 (n = 12; 2.62%), SIT64/LAM6 (n = 8; 1.75%), SIT4014/X1 (n = 7; 1.53%), SIT4015/Unknown (n = 7; 1.53%), SIT283/H1 (n = 6; 1.31%), SIT720/LAM3 (n = 6; 1.31%), SIT1277/LAM9 (n = 6; 1.31%), SIT47/H1 (n = 5; 1.09%), SIT60/LAM4 (n = 5; 1.09%) and SIT91/X3) (n = 5; 1.09%) (**Table 2**).

**Table 2:** Description of clusters containing >1% (n = 5 or more isolates) in this study, and their worldwide distribution in the SITVIT2 database.

SIT (Lineage) Octal NumberSpoligotype Description	Number (%) in study	% in study vs. database	Distribution in Regions with ≥ 3%of a given SIT *	Distribution in countries with ≥ 3%of a given SIT **
**33 (LAM3) 776177607760771** **⬛⬛⬛⬛⬛⬛⬛⬛⬜⬜⬜⬛⬛⬛⬛⬛⬛⬛⬛⬛⬜⬜⬜⬜⬛⬛⬛⬛⬛⬛⬛⬛⬜⬜⬜⬜⬛⬛⬛⬛⬛⬛⬛**	44 (9.61)	3.25	AMER-S 37.08, AFRI-S 24.0, AMER-N 11.74, EURO-S 10.49, EURO-W 6.06, AMER-C 3.99	ZAF 24.0, PER 16.4, USA 11.74, BRA 10.56, ESP 6.57,ARG 4.21, CHL 4.06, FXX 3.91, ITA 3.4, HND 3.18
**37 (T3) 777737777760771** **⬛⬛⬛⬛⬛⬛⬛⬛⬛⬛⬛⬛⬜⬛⬛⬛⬛⬛⬛⬛⬛⬛⬛⬛⬛⬛⬛⬛⬛⬛⬛⬛⬜⬜⬜⬜⬛⬛⬛⬛⬛⬛⬛**	23 (5.02)	4.23	AFRI-E 18.38, AMER-S 13.05, EURO-N 11.4, EURO-W 11.03, ASIA-W 10.11, AMER-N 8.27, ASIA-E 5.88, EURO-S 5.52,EURO-E 4.23, ASIA-S 3.49, AFRI-S 3.13	ETH 15.99, USA 7.35, CHL 5.52, SWE 4.96, SAU 4.78, CHN 4.78, FXX 4.04, ITA 3.86, BRA 3.49, ZAF 3.13, DNK 3.13
**42 (LAM9) 777777607760771** **⬛⬛⬛⬛⬛⬛⬛⬛⬛⬛⬛⬛⬛⬛⬛⬛⬛⬛⬛⬛⬜⬜⬜⬜⬛⬛⬛⬛⬛⬛⬛⬛⬜⬜⬜⬜⬛⬛⬛⬛⬛⬛⬛**	43 (9.39)	1.21	AMER-S 31.99, AMER-N 11.19, EURO-S 10.68, EURO-W 8.94, AFRI-N 8.09, EURO-N 4.62, CARI 4.0, AMER-C 3.35, AFRI-E 3.35	BRA 12.91, USA 11.19, COL 7.19, MAR 6.65, ITA 6.17,FXX 4.76, PER 3.5, ESP 3.16, VEN 3.13
**47 (H1) 777777774020771** **⬛⬛⬛⬛⬛⬛⬛⬛⬛⬛⬛⬛⬛⬛⬛⬛⬛⬛⬛⬛⬛⬛⬛⬛⬛⬜⬜⬜⬜⬜⬜⬛⬜⬜⬜⬜⬛⬛⬛⬛⬛⬛⬛**	5 (1.09)	0.3	EURO-W 19.03, AMER-S 17.06, AMER-N 14.54, EURO-S 12.63, EURO-N 9.81, EURO-E 6.76, ASIA-W 3.77, AFRI-N 3.41	USA 14.24, ITA 7.72, PER 7.54, AUT 7.54, BRA 7.12,FXX 6.22, FIN 5.63, CZE 3.53, ESP 3.35, SWE 3.17
**50 (H3) 777777777720771** **⬛⬛⬛⬛⬛⬛⬛⬛⬛⬛⬛⬛⬛⬛⬛⬛⬛⬛⬛⬛⬛⬛⬛⬛⬛⬛⬛⬛⬛⬛⬜⬛⬜⬜⬜⬜⬛⬛⬛⬛⬛⬛⬛**	27 (5.9)	0.68	AMER-S 26.58, EURO-W 14.88, AMER-N 14.88, EURO-S 9.78, CARI 4.93, EURO-E 4.68, EURO-N 4.63, AFRI-N 3.6,AFRI-S 3.43, AFRI-M 3.2	USA 14.85, PER 13.55, BRA 7.1, FXX 5.83, AUT 5.15,ITA 4.6, ESP 4.6, ZAF 3.43, CMR 3.15, CZE 3.1
**53 (T1) 777777777760771** **⬛⬛⬛⬛⬛⬛⬛⬛⬛⬛⬛⬛⬛⬛⬛⬛⬛⬛⬛⬛⬛⬛⬛⬛⬛⬛⬛⬛⬛⬛⬛⬛⬜⬜⬜⬜⬛⬛⬛⬛⬛⬛⬛**	53 (11.57)	0.8	AMER-S 15.03, EURO-W 14.88, AMER-N 12.82, EURO-S 8.95, EURO-N 7.12, ASIA-W 6.95, AFRI-S 4.72, AFRI-E 4.42, ASIA-E 4.06, AFRI-N 3.35, EURO-E 3.1, CARI 3.07, AMER-C 3.07	USA 12.55, FXX 7.49, BRA 5.57, ITA 5.07, ZAF 4.62,PER 3.71, TUR 3.3, AUT 3.26
**60 (LAM4) 777777607760731** **⬛⬛⬛⬛⬛⬛⬛⬛⬛⬛⬛⬛⬛⬛⬛⬛⬛⬛⬛⬛⬜⬜⬜⬜⬛⬛⬛⬛⬛⬛⬛⬛⬜⬜⬜⬜⬛⬛⬛⬜⬛⬛⬛**	5 (1.09)	1.12	AFRI-S 36.63, AMER-S 24.49, EURO-W 6.97, AFRI-N 6.74,AFRI-W 6.07, EURO-S 5.39, AMER-N 4.27	ZAF 36.63, BRA 14.16, FXX 4.94, USA 4.27, MAR 4.27,GMB 3.82, ITA 3.6, VEN 3.37, PER 3.15
**64 (LAM6) 777777607560771** **⬛⬛⬛⬛⬛⬛⬛⬛⬛⬛⬛⬛⬛⬛⬛⬛⬛⬛⬛⬛⬜⬜⬜⬜⬛⬛⬛⬛⬜⬛⬛⬛⬜⬜⬜⬜⬛⬛⬛⬛⬛⬛⬛**	8 (1.75)	1.94	AMER-S 54.0, AMER-N 22.76, EURO-W 5.81, EURO-S 4.36	BRA 40.44, USA 22.76, GUF 5.81, PRT 3.39
**91 (X3) 700036777760771** **⬛⬛⬛⬜⬜⬜⬜⬜⬜⬜⬜⬜⬜⬛⬛⬛⬛⬜⬛⬛⬛⬛⬛⬛⬛⬛⬛⬛⬛⬛⬛⬛⬜⬜⬜⬜⬛⬛⬛⬛⬛⬛⬛**	5 (1.09)	1.36	AMER-S 37.88, AMER-N 34.33, CARI 17.98, EURO-S 4.36, EURO-N 3.0	USA 32.15, PER 27.79, HTI 16.35, ESP 4.36, COL 3.27
**211 (LAM3) 776137607760771⬛⬛⬛⬛⬛⬛⬛⬛⬜⬜⬜⬛⬜⬛⬛⬛⬛⬛⬛⬛⬜⬜⬜⬜⬛⬛⬛⬛⬛⬛⬛⬛⬜⬜⬜⬜⬛⬛⬛⬛⬛⬛⬛**	12 (2.62)	10.17	AMER-S 27.12, AMER-N 27.12, AMER-C 18.64,EURO-W 17.8, EURO-S 6.78	USA 27.12, MEX 18.64, FXX 16.95, CHL 12.71,BRA 10.17, ESP 4.24
**283 (H1) 777777704020771⬛⬛⬛⬛⬛⬛⬛⬛⬛⬛⬛⬛⬛⬛⬛⬛⬛⬛⬛⬛⬛⬜⬜⬜⬛⬜⬜⬜⬜⬜⬜⬛⬜⬜⬜⬜⬛⬛⬛⬛⬛⬛⬛**	6 (1.31)	8.11	EURO-N 25.68, EURO-W 20.27, AMER-S 18.92, ASIA-S 10.81, AMER-N 8.11, EURO-S 5.41, EURO-E 4.05, AUST 4.05	LVA 18.92, IND 9.46, AUT 9.46, USA 8.11, CHL 8.11, PER 6.76, FXX 6.76, SWE 5.41, NLD 4.05, CZE 4.05, AUS 4.05
**720 (LAM3) 776177607760371⬛⬛⬛⬛⬛⬛⬛⬛⬜⬜⬜⬛⬛⬛⬛⬛⬛⬛⬛⬛⬜⬜⬜⬜⬛⬛⬛⬛⬛⬛⬛⬛⬜⬜⬜⬜⬜⬛⬛⬛⬛⬛⬛**	6 (1.31)	54.55	AMER-S 100.0	CHL 54.55, ARG 36.36, PER 9.09
**1277 (LAM9) 777777207760771⬛⬛⬛⬛⬛⬛⬛⬛⬛⬛⬛⬛⬛⬛⬛⬛⬛⬛⬜⬛⬜⬜⬜⬜⬛⬛⬛⬛⬛⬛⬛⬛⬜⬜⬜⬜⬛⬛⬛⬛⬛⬛⬛**	6 (1.31)	16.67	AMER-S 47.22, CARI 22.22, AMER-N 8.33,AMER-C 8.33, EURO-S 5.56	DOM 22.22, CHL 22.22, USA 8.33, MEX 8.33, BRA 8.33,PER 5.56, ESP 5.56, ARG 5.56
**4014 (X1) 703776777760771⬛⬛⬛⬜⬜⬜⬜⬛⬛⬛⬛⬛⬛⬛⬛⬛⬛⬜⬛⬛⬛⬛⬛⬛⬛⬛⬛⬛⬛⬛⬛⬛⬜⬜⬜⬜⬛⬛⬛⬛⬛⬛⬛**	7 (1.53)	100	AMER-S 100.0	CHL 100.0
**4015 (Unknown) 776177703770371⬛⬛⬛⬛⬛⬛⬛⬛⬜⬜⬜⬛⬛⬛⬛⬛⬛⬛⬛⬛⬛⬜⬜⬜⬜⬛⬛⬛⬛⬛⬛⬛⬛⬜⬜⬜⬜⬛⬛⬛⬛⬛⬛**	7 (1.53)	100	AMER-S 100.0	CHL 100.0

* Worldwide distribution is reported for regions with more than 3% of a given SITs as compared to their total number in the SITVIT2 database. The definition of macro-geographical regions and sub-regions (http://unstats.un.org/unsd/methods/m49/m49regin.htm) is according to the United Nations; Regions: AFRI (Africa), AMER (Americas), ASIA (Asia), EURO (Europe), and OCE (Oceania), subdivided in: E (Eastern), M (Middle), C (Central), N (Northern), S (Southern), SE (South-Eastern), and W (Western). Furthermore, CARIB (Caribbean) belongs to Americas, while Oceania is subdivided in 4 sub-regions, AUST (Australasia), MEL (Melanesia), MIC (Micronesia), and POLY (Polynesia). Note that in our classification scheme, Russia has been attributed a new sub-region by itself (Northern Asia) instead of including it among rest of the Eastern Europe. It reflects its geographical localization as well as due to the similarity of specific TB genotypes circulating in Russia (a majority of Beijing genotypes) with those prevalent in Central, Eastern and South-Eastern Asia.

** The 3 letter country codes are according to http://en.wikipedia.org/wiki/ISO_3166-1_alpha-3; countrywide distribution is only shown for SITs with ≥3% of a given SITs as compared to their total number in the SITVIT2 database. Note that FXX code designates Metropolitan France.

It is noteworthy that SIT33/LAM3 was particularly well represented in our study (n = 44; 9.61%). Also, SIT720/LAM3 was observed in the Chilean population with 6 isolates in our study representing around 54.55% of isolates recorded in SITVIT2 database; this SIT was reported exclusively in South America (see **Table 2**). The other two SITs that were well represented in this study as compared to the registered SITs in SITVIT2 databases, were SIT211 and SIT1277 (10.17% and 16.67% respectively). Interestingly, results obtained from our study suggest that the LAM3 sublineage is very well represented in the Chilean cohort. Interestingly two novel SITs, 4014 and 4015, have been reported at a relatively high proportion (n = 7 isolates each) for the first time in this study.

When the proportion of predominant SITs found in Chile (n = 458 isolates) as well as the distribution patterns for major cities (Santiago, Iquique and Concepción) were compared, it was observed that the strains belonging to SIT91/X3 were localized to Iquique, whereas the strains belonging to SIT37/T3, SIT60/LAM4, SIT64/LAM6, SIT283/H1 and SIT720/LAM3 were mostly isolated from the Santiago based population, and strains belonging to newly created SIT4014/X1 were predominantly found in Concepción city (p-value<0.008; **Table 3**). Significant differences were noted when the distribution patterns of predominant SITs in this study were compared with that of neighboring countries (Peru, Brazil, Paraguay, Argentina) as recorded in the SITVIT2 database (n = 7378 isolates; p-value<0.00001; **Table 3** and **Fig 1A**). In comparison to neighboring countries, SIT33 is well represented in Chile (9.61% of isolates) as compared to others countries. Similar results have also been reported in case of SIT211 and other SITs such as SIT37, SIT64, SIT91, SIT283, SIT720, SIT1277, SIT4014 and SIT4015, which are yet to be reported or present in very low percentages. On the other hand, SIT53, SIT50 and SIT42, which are well represented in our study (11.57%, 5.90%, 9.39% respectively) have also been reported with a relative high percentage in the neighboring countries. In general, it can be concluded that last three SITs are widely distributed. All the analysis described is collated in a phylogeographical distribution map (**Fig 1A**) of major *M*. *tuberculosis* lineages in the three of the most important Chilean cities enrolled in this study (Santiago, Iquique and Concepción), as well as the neighboring countries (Brazil, Paraguay and Peru; data extracted from the SITVIT2 database).

**Table 3:** A comparison of the proportion of predominant SITs found in Chile (n = 458 isolates in total, as well as distribution patterns for major cities, i.e., Santiago, Iquique, and Concepción), as compared to neighboring countries (Peru, Brazil, Paraguay, Argentina), recorded in the SITVIT2 database.

Major M. tuberculosis lineages in the 3 most important cities in our study (Santiago, Iquique, Concepción)							Comparison with neighboring countries available in SITVIT2 database (n = 7378 isolates)							
SIT(Lineage)	Iquique (n = 34)	Santiago (n = 181)	Concepción (n = 34)	Other cities (n = 299)	This study (n = 458)	%	PERU (n = 1282)	%	BRAZIL (n = 4489)	%	PARAGUAY (n = 226)	%	ARGENTINA (n = 1381)	%
**33 (LAM3)**	2	23	3	16	44	9,61	56	4,37	132	2,94	2	0,88	57	4,13
**37 (T3)**	0	14	0	9	23	5,02	0	0,00	10	0,22	0	0,00	0	0,00
**42 (LAM9)**	2	11	1	29	43	9,39	73	5,69	401	8,93	40	17,70	63	4,56
**47 (H1)**	1	3	0	1	5	1,09	40	3,12	110	2,45	2	0,88	8	0,58
**50 (H3)**	1	13	0	13	27	5,90	146	11,39	239	5,32	9	3,98	29	2,10
**53 (T1)**	4	16	2	31	53	11,57	171	13,34	322	7,17	6	2,65	64	4,63
**60 (LAM4)**	0	3	1	1	5	1,09	2	0,16	58	1,29	2	0,88	2	0,14
**64 (LAM6)**	1	5	0	2	8	1,75	7	0,55	138	3,07	0	0,00	4	0,29
**91 (X3)**	3	0	0	2	5	1,09	25	1,95	4	0,09	0	0,00	3	0,22
**211 (LAM3)**	0	4	2	6	12	2,62	1	0,08	10	0,22	1	0,44	1	0,07
**283 (H1)**	0	4	0	2	6	1,31	1	0,08	1	0,02	0	0,00	2	0,14
**720 (LAM3)**	0	5	0	1	6	1,31	1	0,08	0	0,00	0	0,00	4	0,29
**1277 (LAM9)**	1	3	0	2	6	1,31	0	0,00	3	0,07	0	0,00	2	0,14
**4014 (X1)**	0	1	4	2	7	1,53	0	0,00	0	0,00	0	0,00	0	0,00
**4015 (Unknown)**	0	2	2	3	7	1,53	0	0,00	0	0,00	0	0,00	0	0,00

The **Fig 1B** shows the spoligotyping-based MST; it is evident that LAM and T lineages compose two of the most predominant groups in our study and include most of the isolates with frequently observed patterns SIT33, SIT42, and SIT53, SIT37 respectively. Other isolates were classified as the Haarlem (SIT50 recorded as most frequent) and X (SIT4014 recorded as most frequent) lineages. More distance was observed amongst isolates that join in with the Haarlem than those integrating with LAM or T lineages.

**Fig 2:**
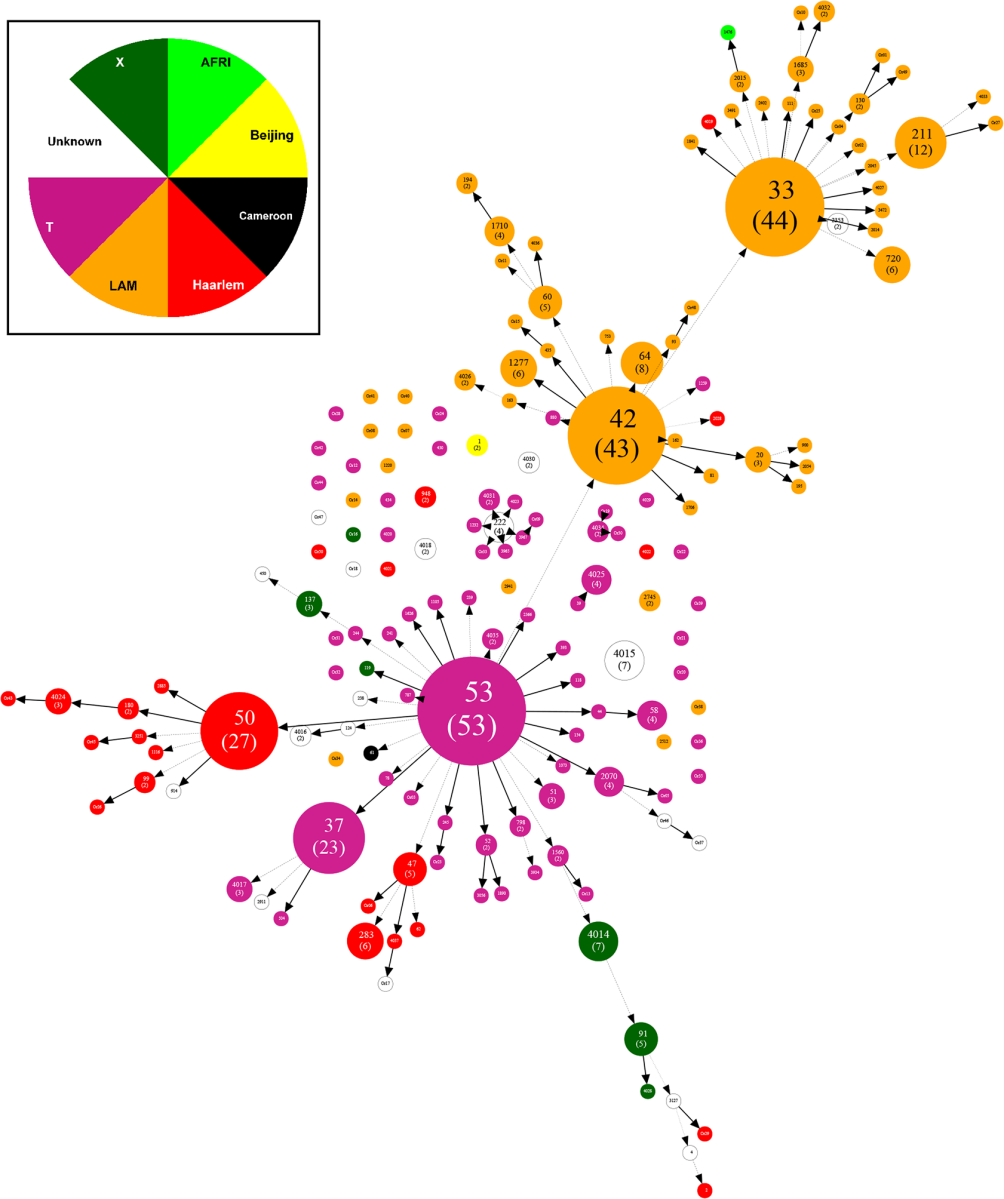
Spoligoforest tree based on all spoligotypes (n = 458 isolates). Spoligoforest was drawn using the Fruchterman-Reingold algorithm from the SpolTools software (http://www.emi.unsw.edu.au/spolTools)[29], and reshaped and colored using the GraphViz software (http://www.graphviz.org)[30]. Each spoligotype pattern from the study is represented by a node with area size being proportional to the total number of isolates with that specific pattern. Changes (loss of spacers) are represented by directed edges between nodes, with the arrowheads pointing to descendant spoligotypes. The heuristic used selects a single inbound edge with a maximum weight using a Zipf model. Solid black lines link patterns that are very similar, i.e., loss of one spacer only (maximum weigh being 1.0), while dashed lines represent links of weight comprised between 0.5 and 1, and dotted lines a weight less than 0.5.

The spoligotype analysis of common SITs (SIT42, SIT53, SIT33, SIT50 and SIT37) clearly demonstrated that they were composed of very closely related isolates and were distinguished from one another by changes in only few alleles/spacers. The spoligoforest tree generated by means of the Fruchterman-Reingold algorithm is illustrated in **Fig 2**. It allows the visualization of all genetic associations and mutations in our strain sample. In this tree, each node represents a spoligotype colored according to the lineage involved, while any potential mutation event that might have occurred from the parental spoligotype is represented as an edge. Thus spoligoforest analysis not only allowed to identify genetic associations between different MTB strains but also helped to gain insight into their genetic diversity and evolution as follows. Briefly, it confirmed the dominance of SIT42, SIT33 (LAM) and SIT53 (T), and to a lesser extent that of SIT50 (H) and SIT37 (T). The SIT42/LAM9 cluster was the largest node evolved from SIT53/T1 and multiple spoligotypes were observed to be rising from it, e.g., SIT33/LAM3, while the second largest spoligotype pattern SIT50/H3 appeared to have derived originally from SIT53/T1. Lastly, the newly created SIT4014/X1 (found only in this study) was a relative precursor of SIT91/X3 (predominantly found in USA, Peru, and Haiti).

## Discussion

In this study, we analyzed the genetic diversity of MTB isolates obtained from clinical samples encompassing 15 regions of Chile. To the best of our knowledge, this is the first study of its kind aimed at investigating the genetic diversity of MTB at the level of the entire country. Our results indicated that the LAM (40.61%), T (34.06%) and Haarlem (13.54%) genotypes were the most abundant spoligotypes of all of *M*. *tuberculosis* samples collected and analyzed. Together, these three lineage families accounted for 88.21% of all genotypes while all other families, namely Beijing, X, AFRI and Cameroon, accounted for only 4.81%. Interestingly, it was noted that the X lineage is poorly represented in the Santiago population (1.66%) whereas in Iquique and Concepción it accounts for nearly 11.76% each. This difference can potentially be explained as a result of: (i) immigration patterns, which have greatly increased in recent years from countries like Peru, Colombia or Ecuador, or (ii) a genetic predisposition that makes the population of these cities more susceptible to certain genotypes.

This is consistent with data available from most South American countries which claim that the most represented lineages are LAM, T and Haarlem in proportions that are similar to those reported by us; in some cases the proportions may be subject to change but invariably it is these three families that dominate epidemiologically. In **Fig 1A**, a representation of data available from three of our neighboring countries (Peru, Brazil and Paraguay) and three of the major Chilean cities included in this study are plotted. Several published reports on population structure of MTB in South America have emphatically proven that each family is adequately represented from an epidemiological perspective [14–22]. In Brazil, Vasconcellos and collaborators demonstrated via spoligotyping that LAM is represented in 43.6%, T in 34.9% and Haarlem 18.3% of case studies. Similar results were also found in a study where isolates from patients belonging to 11 states of Brazil were analyzed. The mentioned study demonstrated that the predominant MTB lineage was LAM (46%) followed by T (18.6%), Haarlem (12.2%), X (4.7%), S (1.9%) and, lastly, the East African Indian (EAI) (0.85%) families [14]. However, in Peru and Bolivia it is reported that the most represented family is Haarlem, followed by the LAM and T families [20,33,34]. For Argentina there is comparatively less information in literature but available data clearly show that Haarlem is predominant except for MDR cases where the LAM family dominates [35,36]. In a recent work that was conducted in the border areas of Brazil, Argentina and Paraguay, a higher presence of the LAM family was reported [37]. All of the above mentioned variance can be potentially accounted for by: i) number of isolates analyzed, ii) region of sampling (restricted to a small region or spanning all country) iii) year of sampling, etc.

Fifteen SITs were noted to be the most represented (n = 5 or more isolates) in this study (**Table 2**). SIT53/T1 was the most prevalent suggesting that this particular lineage is actively circulating in Chile. This finding is corroborated by findings regarding the worldwide scene which also states that this particular SIT is widely distributed (**Table 2**) [22]. SIT33/LAM3 (9.61%) and SIT42/LAM9 (9.39%) are present in Brazil in proportions similar to reported by us but in Peru and Paraguay the presence of these two SITs is only about 3% (**Table 3**). **Fig 1B** represents the main SITs obtained in this study (labeled circle) as well as the genetic evolution based on distance and allele/spacer change between patterns. In case of the MST, it is possible to ascertain major evolutionary pathways followed by the MTB lineages by observing the similarity between each strain. Further analysis leads us to observe that the Beijing family group is very far from the Euro American lineage present in the central nodes. We also concluded that the strains belonging to the LAM lineage have a high genetic diversity and that the T and Haarlem lineages are genetically closer.

Three additional published studies have analyzed the genetic diversity of MTB in Chile [25–27]. Although all three studies were limited by the fact that they were restricted to a single region of the country; they unanimously concluded that the LAM family was the most predominant lineage in Chile. A recently published work [27] showed that almost two-thirds of the circulating strains in Santiago city could be accounted for by the prevalence of LAM and T genotypes. The most frequent SITs that were seen to cluster spoligotypes were SIT33/LAM3 (10.7%), SIT53/T1 (8.7%), SIT50/H3 (7.8%) and SIT37/T3 (6.8%). The distribution pattern in the above mentioned study goes in parallel with data obtained in our study. Last but not least, our study further corroborated the high presence of SIT37/T3 (n = 23) in the Chilean population, a pattern that was usually found in Eastern Africa in the international database.

Currently, the National Control Program of Human Tuberculosis in Chile [13] has managed to position our country among nations that have managed to control this disease. The incidence of TB in Chile, when compared to other South American countries, is low at only 12.5 per 100,000. During the 2009–2013 period, the incidence value for Brazil was of 46, 44 in Paraguay and 124 in Peru (per 100,000). The situation in Peru, a neighboring country with which we share the border, attracts our attention because the differences in incidence rates are so overwhelming. This could be a possible effect of immigration and public health policies. As with other airborne diseases, the spread of MTB is facilitated by high population densities and crowded indoor environments that optimize the transmission of the pathogen. Other host-related factors that are implicated in increasing the chances of infection are: (i) immune suppression, (ii) smoking, (iii) poor nutrition, (iv) diabetes, and (v) respiratory comorbidities. All these factors can also result in an increase in the risk of transitioning from latent to active MTB [38]. This is a very important issue in the developing world where overcrowding, malnutrition and HIV infection contribute to a high burden of the disease.

Drug resistant tests conducted as part of our study revealed that 87.7% (n = 402) of the isolates were pan-susceptible, 27.8% (n = 44) were resistant to any drugs, and 1.31% (n = 6) were MDR; for 1.31% (n = 6 isolates) the data could not be obtained. Three of the six strains that presented with multi drug resistance belonged to SIT53/T1. Conversely, LAM (which is the most prevalent lineage in our study), was apparently overrepresented among MDR cases in French departments of the Americas–based on data collection over an extended period of time [39], as well as in Peru [40]. Lastly, half of the SIT4015 isolates (of a lineage with unknown signature) showed drug resistance. This pattern should be carefully surveyed in coming years to see if its emergence is not linked to specific host factors since different ethnic groups may have varying susceptibilities to various MTB strains [41]. Indeed, both SIT4014/X1 (n = 7 isolates) and SIT4015 (Unknown signature; n = 7 isolates) have only been found in Chile so far. Considering that most of the samples belonging to these SITs were collected in the city of Concepcion and its vicinity, one should take into account associated factors such as susceptibility genetics of the host when studying emerging MTB clones. A similar case can be made for SIT720/LAM3, which is so far restricted to South America. In summary, our results underline the importance of tracking circulating and emerging MTB clones in conjunction with development of phylogenetical databases in order to maintain proper vigilance. These measures are a powerful way to safeguard against possible future outbreaks.

Although Spoligotyping can contribute to identifying outbreaks and tracking the spread of disease, it has been known to overestimate clustering of isolates. Therefore, it is our belief that a more polymorphic technique such as MIRU-VNTR technique would be able to impart greater in-depth information that will help improve our knowledge of current epidemiological data in Chile.

To conclude, in this study, spanning 15 regions of Chile, we have shown that the LAM, T, and Haarlem lineages together represent 88.2% of the isolates. This corroborates that the data obtained from the neighboring countries also show that the Euro-American lineages have a clear epidemiological superiority. The exclusive emergence of SIT4014 and SIT4015 indicates that conditions specific to Chile, along with the unique genetic makeup of the Chilean population, might have allowed for a possible co-evolution of certain genotypes leading to their relative success to cause disease.

## Supporting Information

S1 Fig Geographic distribution of isolates collected within of 15 regions of Chile (n = 458 strains). (TIF)

S1 Table Detailed demographic and genotyping information on *M*. *tuberculosis* isolates from Chile (n = 458 strains). (PDF)
