# Supplementary figures and images for: Analysis of Mycobacterium tuberculosis Genotypic Lineage Distribution in Chile and Neighboring Countries
Source: PLoS One. 2016 Aug 12;11(8):e0160434. doi: 10.1371/journal.pone.0160434 (PMC4982630; doi:10.1371/journal.pone.0160434)

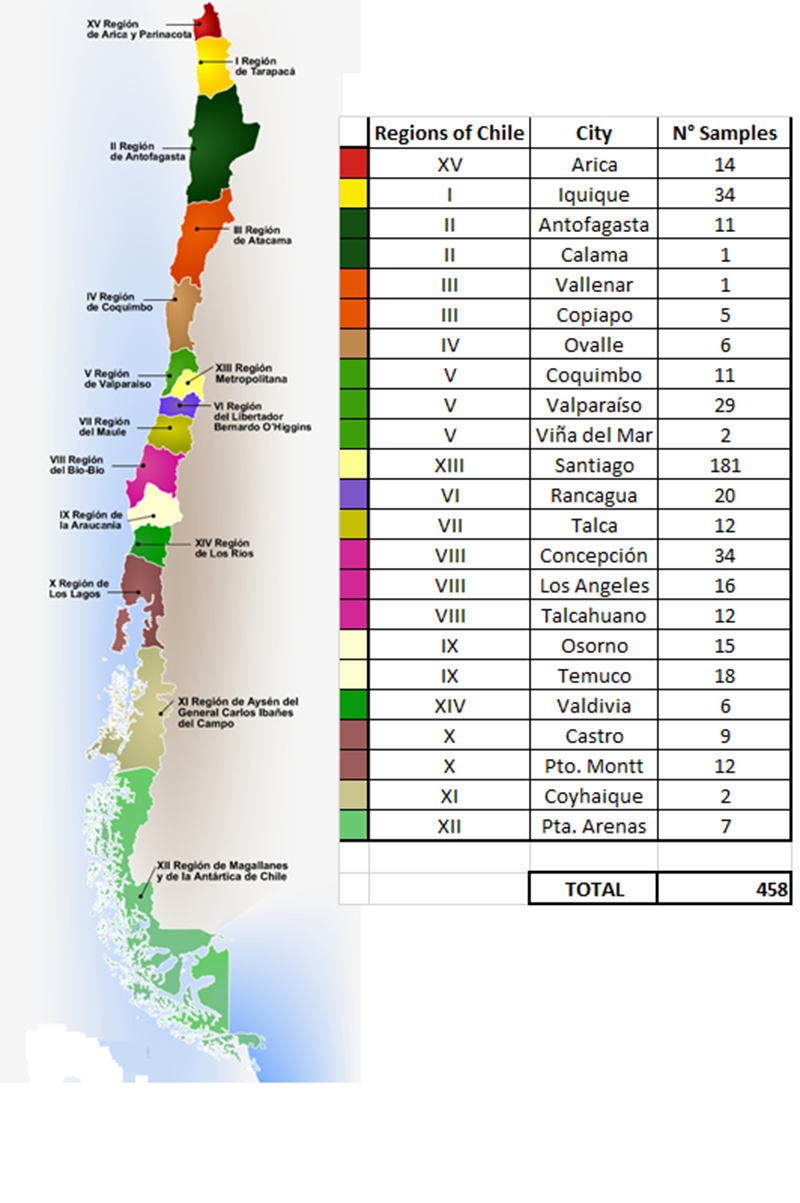

Supplement: S1 Fig — (TIF) [file pone.0160434.s001.tif]
