# Supplementary material for: Analysis of Mycobacterium tuberculosis Genotypic Lineage Distribution in Chile and Neighboring Countries
Source: PLoS One. 2016 Aug 12;11(8):e0160434. doi: 10.1371/journal.pone.0160434 (PMC4982630; doi:10.1371/journal.pone.0160434)
Supplement: S1 Table — (PDF) [file pone.0160434.s002.pdf]

Supplemental Table S1: Detailed demographic and genotyping information on *M. tuberculosis* isolates from Chile (n=458 strains).

Strain Number	Year	Sex	Isolation city	Spoligotype Description	Octal code	SIT*	Lineage	Drug resistance**
2035-2011-S-1414	2011	Male	Iquique	□□□□□□□□□□□□□□□□□□□□□□□□□□□□□□□□□□■■■■■■■■■	000000000003771	1	Beijing	Pansusceptible
406-2012-S-288	2012	Male	Santiago	□□□□□□□□□□□□□□□□□□□□□□□□□□□□□□□□□□■■■■■■■■■	000000000003771	1	Beijing	Pansusceptible
283-2012-S-215	2012	Male	Valparaiso	□□□□□□□□□□□□□□□□□□□□□□□□■□□□□□□■□□□□■■■■■■■	000000004020771	2	H2	Pansusceptible
2060-2011-S-1435	2011	Male	Coquimbo	□□□□□□□□□□□□□□□□□□□□□□□□■■■■■■■■□□□□■■■■■■■	000000007760771	4	Unknown	Pansusceptible
1758-2011-S-1173	2011	Female	Rancagua	■■□■■■■■■■■■■■■■■■■■□□□□■■■■■■■■□□□□■■■■■■■	677777607760771	20	LAM1	Pansusceptible
1831-2011-S-1267	2011	Male	Santiago	■■□■■■■■■■■■■■■■■■■■□□□□■■■■■■■■□□□□■■■■■■■	677777607760771	20	LAM1	R (SM)
829-2011-S-505	2011	Female	Puerto Montt	■■□■■■■■■■■■■■■■■■■■□□□□■■■■■■■■□□□□■■■■■■■	677777607760771	20	LAM1	Pansusceptible
1000-2012-S-632	2012	Male	Los Angeles	■■■■■■■■□□□■■■■■■■■■□□□□■■■■■■■■□□□□■■■■■■■	776177607760771	33	LAM3	Pansusceptible
1013-2011-S-629	2011	Female	Santiago	■■■■■■■■□□□■■■■■■■■■□□□□■■■■■■■■□□□□■■■■■■■	776177607760771	33	LAM3	Pansusceptible
1035-2012-S-655	2012	Male	Santiago	■■■■■■■■□□□■■■■■■■■■□□□□■■■■■■■■□□□□■■■■■■■	776177607760771	33	LAM3	Pansusceptible
1097-2011-S-687	2011	Female	Talcahuano	■■■■■■■■□□□■■■■■■■■■□□□□■■■■■■■■□□□□■■■■■■■	776177607760771	33	LAM3	Pansusceptible
1101-2011-S-690	2011	Male	Santiago	■■■■■■■■□□□■■■■■■■■■□□□□■■■■■■■■□□□□■■■■■■■	776177607760771	33	LAM3	R (SM)
1139-2011-S-718	2011	Female	Santiago	■■■■■■■■□□□■■■■■■■■■□□□□■■■■■■■■□□□□■■■■■■■	776177607760771	33	LAM3	Pansusceptible
1187-2011-S-751	2011	Male	Santiago	■■■■■■■■□□□■■■■■■■■■□□□□■■■■■■■■□□□□■■■■■■■	776177607760771	33	LAM3	Pansusceptible
1208-2011-S-763	2011	Female	Santiago	■■■■■■■■□□□■■■■■■■■■□□□□■■■■■■■■□□□□■■■■■■■	776177607760771	33	LAM3	Pansusceptible
1231-2011-S-781	2011	Male	Santiago	■■■■■■■■□□□■■■■■■■■■□□□□■■■■■■■■□□□□■■■■■■■	776177607760771	33	LAM3	Pansusceptible
1263-2011-S-796	2011	Female	Iquique	■■■■■■■■□□□■■■■■■■■■□□□□■■■■■■■■□□□□■■■■■■■	776177607760771	33	LAM3	R (SM)
1307-2011-S-830	2011	Male	Santiago	■■■■■■■■□□□■■■■■■■■■□□□□■■■■■■■■□□□□■■■■■■■	776177607760771	33	LAM3	Pansusceptible
1319-2011-S-833	2011	Female	Los Angeles	■■■■■■■■□□□■■■■■■■■■□□□□■■■■■■■■□□□□■■■■■■■	776177607760771	33	LAM3	Pansusceptible
1354-2011-S-857	2011	Male	Vallenar	■■■■■■■■□□□■■■■■■■■■□□□□■■■■■■■■□□□□■■■■■■■	776177607760771	33	LAM3	Pansusceptible
1370-2011-S-870	2011	Male	Santiago	■■■■■■■■□□□■■■■■■■■■□□□□■■■■■■■■□□□□■■■■■■■	776177607760771	33	LAM3	Pansusceptible
1413-2011-S-903	2011	Female	Santiago	■■■■■■■■□□□■■■■■■■■■□□□□■■■■■■■■□□□□■■■■■■■	776177607760771	33	LAM3	Pansusceptible
1460-2011-S-942	2011	Male	Antofagasta	■■■■■■■■□□□■■■■■■■■■□□□□■■■■■■■■□□□□■■■■■■■	776177607760771	33	LAM3	Pansusceptible
1474-2011-S-951	2011	Male	Santiago	■■■■■■■■□□□■■■■■■■■■□□□□■■■■■■■■□□□□■■■■■■■	776177607760771	33	LAM3	Pansusceptible
1475-2011-S-952	2011	Male	Santiago	■■■■■■■■□□□■■■■■■■■■□□□□■■■■■■■■□□□□■■■■■■■	776177607760771	33	LAM3	R (SM)
1744-2011-S-1159	2011	Male	Rancagua	■■■■■■■■□□□■■■■■■■■■□□□□■■■■■■■■□□□□■■■■■■■	776177607760771	33	LAM3	Pansusceptible
1767-2011-S-1181	2011	Male	Santiago	■■■■■■■■□□□■■■■■■■■■□□□□■■■■■■■■□□□□■■■■■■■	776177607760771	33	LAM3	Pansusceptible
1779-2011-S-1189	2011	Female	Santiago	■■■■■■■■□□□■■■■■■■■■□□□□■■■■■■■■□□□□■■■■■■■	776177607760771	33	LAM3	Pansusceptible
1783-2011-S-1192	2011	Male	Los Angeles	■■■■■■■■□□□■■■■■■■■■□□□□■■■■■■■■□□□□■■■■■■■	776177607760771	33	LAM3	Pansusceptible
1828-2011-S-1264	2011	Male	Santiago	■■■■■■■■□□□■■■■■■■■■□□□□■■■■■■■■□□□□■■■■■■■	776177607760771	33	LAM3	Pansusceptible
1840-2011-S-1275	2011	Male	Castro	■■■■■■■■□□□■■■■■■■■■□□□□■■■■■■■■□□□□■■■■■■■	776177607760771	33	LAM3	Pansusceptible
1857-2011-S-1285	2011	Male	Santiago	■■■■■■■■□□□■■■■■■■■■□□□□■■■■■■■■□□□□■■■■■■■	776177607760771	33	LAM3	R (PZA)
1961-2011-S-1365	2011	Female	Osorno	■■■■■■■■□□□■■■■■■■■■□□□□■■■■■■■■□□□□■■■■■■■	776177607760771	33	LAM3	Pansusceptible
2020-2011-S-1404	2011	Male	Concepcion	■■■■■■■■□□□■■■■■■■■■□□□□■■■■■■■■□□□□■■■■■■■	776177607760771	33	LAM3	Pansusceptible
259-2012-S-201	2012	Male	Santiago	■■■■■■■■□□□■■■■■■■■■□□□□■■■■■■■■□□□□■■■■■■■	776177607760771	33	LAM3	Pansusceptible
304-2012-S-231	2012	Male	Concepcion	■■■■■■■■□□□■■■■■■■■■□□□□■■■■■■■■□□□□■■■■■■■	776177607760771	33	LAM3	Pansusceptible
319-2012-S-238	2012	Male	Coihaique	■■■■■■■■□□□■■■■■■■■■□□□□■■■■■■■■□□□□■■■■■■■	776177607760771	33	LAM3	Pansusceptible
419-2012-S-294	2012	Male	Los Angeles	■■■■■■■■□□□■■■■■■■■■□□□□■■■■■■■■□□□□■■■■■■■	776177607760771	33	LAM3	Pansusceptible
714-2012-S-478	2012	Female	Ovalle	■■■■■■■■□□□■■■■■■■■■□□□□■■■■■■■■□□□□■■■■■■■	776177607760771	33	LAM3	Pansusceptible
736-2012-S-489	2012	Female	Los Angeles	■■■■■■■■□□□■■■■■■■■■□□□□■■■■■■■■□□□□■■■■■■■	776177607760771	33	LAM3	Pansusceptible
743-2012-S-492	2012	Female	Santiago	■■■■■■■■□□□■■■■■■■■■□□□□■■■■■■■■□□□□■■■■■■■	776177607760771	33	LAM3	R (SM)
762-2012-S-505	2012	Male	Punta Arenas	■■■■■■■■□□□■■■■■■■■■□□□□■■■■■■■■□□□□■■■■■■■	776177607760771	33	LAM3	Pansusceptible
820-2012-S-535	2012	Male	Talca	■■■■■■■■□□□■■■■■■■■■□□□□■■■■■■■■□□□□■■■■■■■	776177607760771	33	LAM3	Pansusceptible
822-2012-S-537	2012	Male	Santiago	■■■■■■■■□□□■■■■■■■■■□□□□■■■■■■■■□□□□■■■■■■■	776177607760771	33	LAM3	Pansusceptible
830-2011-S-506	2011	Male	Puerto Montt	■■■■■■■■□□□■■■■■■■■■□□□□■■■■■■■■□□□□■■■■■■■	776177607760771	33	LAM3	Pansusceptible
832-2011-S-507	2011	Male	Santiago	■■■■■■■■□□□■■■■■■■■■□□□□■■■■■■■■□□□□■■■■■■■	776177607760771	33	LAM3	Pansusceptible
875-2011-S-532	2011	Male	Santiago	■■■■■■■■□□□■■■■■■■■■□□□□■■■■■■■■□□□□■■■■■■■	776177607760771	33	LAM3	Pansusceptible
886-2012-S-569	2012	Male	Santiago	■■■■■■■■□□□■■■■■■■■■□□□□■■■■■■■■□□□□■■■■■■■	776177607760771	33	LAM3	Pansusceptible
948-2011-S-581	2011	Male	Concepcion	■■■■■■■■□□□■■■■■■■■■□□□□■■■■■■■■□□□□■■■■■■■	776177607760771	33	LAM3	Pansusceptible
955-2011-S-588	2011	Male	Santiago	■■■■■■■■□□□■■■■■■■■■□□□□■■■■■■■■□□□□■■■■■■■	776177607760771	33	LAM3	Pansusceptible
975-2011-S-599	2011	Male	Iquique	■■■■■■■■□□□■■■■■■■■■□□□□■■■■■■■■□□□□■■■■■■■	776177607760771	33	LAM3	R (SM)
1082-2011-S-676	2011	Male	Temuco	■■■■■■■■■■■■□■■■■■■■■■■■■■■■■■■■□□□□■■■■■■■	777737777760771	37	T3	Pansusceptible
1183-2012-S-745	2012	Female	Santiago	■■■■■■■■■■■■□■■■■■■■■■■■■■■■■■■■□□□□■■■■■■■	777737777760771	37	T3	Pansusceptible
1190-2012-S-751	2012	Male	Santiago	■■■■■■■■■■■■□■■■■■■■■■■■■■■■■■■■□□□□■■■■■■■	777737777760771	37	T3	Pansusceptible
1280-2011-S-807	2011	Male	Santiago	■■■■■■■■■■■■□■■■■■■■■■■■■■■■■■■■□□□□■■■■■■■	777737777760771	37	T3	Pansusceptible
1367-2011-S-867	2011	Male	Temuco	■■■■■■■■■■■■□■■■■■■■■■■■■■■■■■■■□□□□■■■■■■■	777737777760771	37	T3	Pansusceptible
1827-2011-S-1263	2011	Male	Santiago	■■■■■■■■■■■■□■■■■■■■■■■■■■■■■■■■□□□□■■■■■■■	777737777760771	37	T3	Pansusceptible
1844-2011-S-1279	2011	Male	Santiago	■■■■■■■■■■■■□■■■■■■■■■■■■■■■■■■■□□□□■■■■■■■	777737777760771	37	T3	Pansusceptible
1924-2011-S-1337	2011	Male	Santiago	■■■■■■■■■■■■□■■■■■■■■■■■■■■■■■■■□□□□■■■■■■■	777737777760771	37	T3	Pansusceptible
1926-2011-S-1338	2011	Male	Santiago	■■■■■■■■■■■■□■■■■■■■■■■■■■■■■■■■□□□□■■■■■■■	777737777760771	37	T3	Pansusceptible
1985-2011-S-1381	2011	Male	Antofagasta	■■■■■■■■■■■■□■■■■■■■■■■■■■■■■■■■□□□□■■■■■■■	777737777760771	37	T3	Pansusceptible
2008-2011-S-1397	2011	Female	Valparaiso	■■■■■■■■■■■■□■■■■■■■■■■■■■■■■■■■□□□□■■■■■■■	777737777760771	37	T3	Pansusceptible
272-2012-S-211	2012	Female	Puerto Montt	■■■■■■■■■■■■□■■■■■■■■■■■■■■■■■■■□□□□■■■■■■■	777737777760771	37	T3	Pansusceptible
458-2012-S-319	2012	Male	Santiago	■■■■■■■■■■■■□■■■■■■■■■■■■■■■■■■■□□□□■■■■■■■	777737777760771	37	T3	Pansusceptible
513-2012-S-352	2012	Female	Santiago	■■■■■■■■■■■■□■■■■■■■■■■■■■■■■■■■□□□□■■■■■■■	777737777760771	37	T3	Pansusceptible
602-2012-S-410	2012	Male	Valparaiso	■■■■■■■■■■■■□■■■■■■■■■■■■■■■■■■■□□□□■■■■■■■	777737777760771	37	T3	Pansusceptible
677-2012-S-450	2012	Male	Santiago	■■■■■■■■■■■■□■■■■■■■■■■■■■■■■■■■□□□□■■■■■■■	777737777760771	37	T3	Pansusceptible
916-2011-S-559	2011	Male	Santiago	■■■■■■■■■■■■□■■■■■■■■■■■■■■■■■■■□□□□■■■■■■■	777737777760771	37	T3	Pansusceptible
939-2011-S-575	2011	Female	Arica	■■■■■■■■■■■■□■■■■■■■■■■■■■■■■■■■□□□□■■■■■■■	777737777760771	37	T3	Pansusceptible
942-2011-S-577	2011	Female	Arica	■■■■■■■■■■■■□■■■■■■■■■■■■■■■■■■■□□□□■■■■■■■	777737777760771	37	T3	Pansusceptible
952-2011-S-585	2011	Male	Santiago	■■■■■■■■■■■■□■■■■■■■■■■■■■■■■■■■□□□□■■■■■■■	777737777760771	37	T3	Pansusceptible
953-2011-S-586	2011	Male	Santiago	■■■■■■■■■■■■□■■■■■■■■■■■■■■■■■■■□□□□■■■■■■■	777737777760771	37	T3	Pansusceptible
972-2011-S-597	2011	Male	Santiago	■■■■■■■■■■■■□■■■■■■■■■■■■■■■■■■■□□□□■■■■■■■	777737777760771	37	T3	Pansusceptible
993-2012-S-629	2012	Male	Rancagua	■■■■■■■■■■■■□■■■■■■■■■■■■■■■■■■■□□□□■■■■■■■	777737777760771	37	T3	Pansusceptible
1233-2011-S-783	2011	Male	Santiago	■■■■■■■■■■■■■■■■■■□■■■□□■■■■■■■■□□□□■□□■■■■	777777347760471	39	T4-CEU1	Pansusceptible
101-2012-S-89	2012	Male	Rancagua	■■■■■■■■■■■■■■■■■■■■□□□□■■■■■■■■□□□□■■■■■■■	777777607760771	42	LAM9	Pansusceptible
1078-2011-S-672	2011	Female	Temuco	■■■■■■■■■■■■■■■■■■■■□□□□■■■■■■■■□□□□■■■■■■■	777777607760771	42	LAM9	Pansusceptible
1112-2011-S-699	2011	Female	Puerto Montt	■■■■■■■■■■■■■■■■■■■■□□□□■■■■■■■■□□□□■■■■■■■	777777607760771	42	LAM9	Pansusceptible
1141-2011-S-719	2011	Female	Santiago	■■■■■■■■■■■■■■■■■■■■□□□□■■■■■■■■□□□□■■■■■■■	777777607760771	42	LAM9	Pansusceptible
1201-2011-S-760	2011	Male	Puerto Montt	■■■■■■■■■■■■■■■■■■■■□□□□■■■■■■■■□□□□■■■■■■■	777777607760771	42	LAM9	Pansusceptible
1240-2011-S-786	2011	Male	Valparaiso	■■■■■■■■■■■■■■■■■■■■□□□□■■■■■■■■□□□□■■■■■■■	777777607760771	42	LAM9	Pansusceptible
1264-2011-S-797	2011	Male	Iquique	■■■■■■■■■■■■■■■■■■■■□□□□■■■■■■■■□□□□■■■■■■■	777777607760771	42	LAM9	Pansusceptible
1283-2011-S-810	2011	Male	Concepcion	■■■■■■■■■■■■■■■■■■■■□□□□■■■■■■■■□□□□■■■■■■■	777777607760771	42	LAM9	Pansusceptible
1309-2011-S-831	2011	Female	Santiago	■■■■■■■■■■■■■■■■■■■■□□□□■■■■■■■■□□□□■■■■■■■	777777607760771	42	LAM9	Pansusceptible
1321-2011-S-835	2011	Female	Viña del Mar	■■■■■■■■■■■■■■■■■■■■□□□□■■■■■■■■□□□□■■■■■■■	777777607760771	42	LAM9	R (PZA)
1346-2011-S-855	2011	Female	Iquique	■■■■■■■■■■■■■■■■■■■■□□□□■■■■■■■■□□□□■■■■■■■	777777607760771	42	LAM9	Pansusceptible
136-2012-S-118	2012	Female	Santiago	■■■■■■■■■■■■■■■■■■■■□□□□■■■■■■■■□□□□■■■■■■■	777777607760771	42	LAM9	Pansusceptible
1365-2011-S-865	2011	Male	Valdivia	■■■■■■■■■■■■■■■■■■■■□□□□■■■■■■■■□□□□■■■■■■■	777777607760771	42	LAM9	Pansusceptible
1366-2011-S-866	2011	Female	Temuco	■■■■■■■■■■■■■■■■■■■■□□□□■■■■■■■■□□□□■■■■■■■	777777607760771	42	LAM9	Pansusceptible
1372-2011-S-872	2011	Male	Santiago	■■■■■■■■■■■■■■■■■■■■□□□□■■■■■■■■□□□□■■■■■■■	777777607760771	42	LAM9	Pansusceptible
1420-2011-S-910	2011	Male	Santiago	■■■■■■■■■■■■■■■■■■■■□□□□■■■■■■■■□□□□■■■■■■■	777777607760771	42	LAM9	R (PZA)
1461-2011-S-943	2011	Male	Temuco	■■■■■■■■■■■■■■■■■■■■□□□□■■■■■■■■□□□□■■■■■■■	777777607760771	42	LAM9	Pansusceptible
1496-2011-S-970	2011	Male	Valparaiso	■■■■■■■■■■■■■■■■■■■■□□□□■■■■■■■■□□□□■■■■■■■	777777607760771	42	LAM9	Pansusceptible
1497-2011-S-971	2011	Male	Valparaiso	■■■■■■■■■■■■■■■■■■■■□□□□■■■■■■■■□□□□■■■■■■■	777777607760771	42	LAM9	Pansusceptible
156-2012-S-128	2012	Male	Talcahuano	■■■■■■■■■■■■■■■■■■■■□□□□■■■■■■■■□□□□■■■■■■■	777777607760771	42	LAM9	R (SM)
1642-2011-S-1085	2011	Male	Antofagasta	■■■■■■■■■■■■■■■■■■■■□□□□■■■■■■■■□□□□■■■■■■■	777777607760771	42	LAM9	Pansusceptible
1733-2011-S-1152	2011	Male	Punta Arenas	■■■■■■■■■■■■■■■■■■■■□□□□■■■■■■■■□□□□■■■■■■■	777777607760771	42	LAM9	Pansusceptible
1750-2011-S-1164	2011	Male	Rancagua	■■■■■■■■■■■■■■■■■■■■□□□□■■■■■■■■□□□□■■■■■■■	777777607760771	42	LAM9	R (PZA)
1853-2011-S-1282	2011	Male	Arica	■■■■■■■■■■■■■■■■■■■■□□□□■■■■■■■■□□□□■■■■■■■	777777607760771	42	LAM9	Pansusceptible
1865-2011-S-1290	2011	Female	Rancagua	■■■■■■■■■■■■■■■■■■■■□□□□■■■■■■■■□□□□■■■■■■■	777777607760771	42	LAM9	Pansusceptible

1866-2011-S-1291	2011	Female	Rancagua	■■■■■■■■■■■■■■■■■■■■□□□□■■■■■■■■□□□□■■■■■■■	777777607760771	42	LAM9	Pansusceptible
1887-2011-S-1307	2011	Male	Valparaiso	■■■■■■■■■■■■■■■■■■■■□□□□■■■■■■■■□□□□■■■■■■■	777777607760771	42	LAM9	Pansusceptible
2028-2011-S-1410	2011	Male	Santiago	■■■■■■■■■■■■■■■■■■■■□□□□■■■■■■■■□□□□■■■■■■■	777777607760771	42	LAM9	Pansusceptible
418-2012-S-293	2012	Male	Los Angeles	■■■■■■■■■■■■■■■■■■■■□□□□■■■■■■■■□□□□■■■■■■■	777777607760771	42	LAM9	Pansusceptible
544-2012-S-366	2012	Male	Puerto Montt	■■■■■■■■■■■■■■■■■■■■□□□□■■■■■■■■□□□□■■■■■■■	777777607760771	42	LAM9	Pansusceptible
563-2012-S-382	2012	Male	Santiago	■■■■■■■■■■■■■■■■■■■■□□□□■■■■■■■■□□□□■■■■■■■	777777607760771	42	LAM9	Pansusceptible
547-2012-S-372	2012	Male	Ovalle	■■■■■■■■■■■■■■■■■■■■□□□□■■■■■■■■□□□□■■■■■■■	777777607760771	42	LAM9	Pansusceptible
700-2012-S-464	2012	Male	Antofagasta	■■■■■■■■■■■■■■■■■■■■□□□□■■■■■■■■□□□□■■■■■■■	777777607760771	42	LAM9	Pansusceptible
760-2012-S-503	2012	Male	Talca	■■■■■■■■■■■■■■■■■■■■□□□□■■■■■■■■□□□□■■■■■■■	777777607760771	42	LAM9	R (INH)
761-2012-S-504	2012	Female	Talca	■■■■■■■■■■■■■■■■■■■■□□□□■■■■■■■■□□□□■■■■■■■	777777607760771	42	LAM9	Pansusceptible
789-2011-S-489	2012	Female	Santiago	■■■■■■■■■■■■■■■■■■■■□□□□■■■■■■■■□□□□■■■■■■■	777777607760771	42	LAM9	Pansusceptible
827-2011-S-504	2011	Female	Temuco	■■■■■■■■■■■■■■■■■■■■□□□□■■■■■■■■□□□□■■■■■■■	777777607760771	42	LAM9	Pansusceptible
885-2011-S-538	2011	Male	Valdivia	■■■■■■■■■■■■■■■■■■■■□□□□■■■■■■■■□□□□■■■■■■■	777777607760771	42	LAM9	Pansusceptible
910-2011-S-553	2011	Female	Santiago	■■■■■■■■■■■■■■■■■■■■□□□□■■■■■■■■□□□□■■■■■■■	777777607760771	42	LAM9	Pansusceptible
912-2011-S-555	2011	Male	Santiago	■■■■■■■■■■■■■■■■■■■■□□□□■■■■■■■■□□□□■■■■■■■	777777607760771	42	LAM9	Pansusceptible
921-2011-S-565	2011	Female	Castro	■■■■■■■■■■■■■■■■■■■■□□□□■■■■■■■■□□□□■■■■■■■	777777607760771	42	LAM9	R (INH+SM)
954-2011-S-587	2011	Male	Santiago	■■■■■■■■■■■■■■■■■■■■□□□□■■■■■■■■□□□□■■■■■■■	777777607760771	42	LAM9	Pansusceptible
99-2012-S-88	2012	Male	Talca	■■■■■■■■■■■■■■■■■■■■□□□□■■■■■■■■□□□□■■■■■■■	777777607760771	42	LAM9	Pansusceptible
1405-2011-S-897	2011	Female	Coquimbo	■■■■■■■■■■■■■■■■■■■■■■□■■■■■■■■■■□□□□■■■■■■■	777777757760771	44	T5	NA
1060-2011-S-661	2011	Female	Santiago	■■■■■■■■■■■■■■■■■■■■■■■■■□□□□□□■□□□□■■■■■■■	777777774020771	47	H1	Pansusceptible
1123-2011-S-708	2011	Male	Valparaiso	■■■■■■■■■■■■■■■■■■■■■■■■■□□□□□□■□□□□■■■■■■■	777777774020771	47	H1	Pansusceptible
1728-2011-S-1151	2011	Male	Santiago	■■■■■■■■■■■■■■■■■■■■■■■■■□□□□□□■□□□□■■■■■■■	777777774020771	47	H1	R (INH)
246-2012-S-193	2012	Male	Santiago	■■■■■■■■■■■■■■■■■■■■■■■■■□□□□□□■□□□□■■■■■■■	777777774020771	47	H1	Pansusceptible
980-2011-S-604	2011	Male	Iquique	■■■■■■■■■■■■■■■■■■■■■■■■■□□□□□□■□□□□■■■■■■■	777777774020771	47	H1	R (PZA)
1051-2011-S-657	2011	Male	Arica	■■■■■■■■■■■■■■■■■■■■■■■■■■■■■■□■□□□□■■■■■■■	777777777720771	50	H3	R (INH+SM)
1176-2011-S-744	2011	Female	Temuco	■■■■■■■■■■■■■■■■■■■■■■■■■■■■■■□■□□□□■■■■■■■	777777777720771	50	H3	Pansusceptible
1221-2011-S-774	2011	Male	Coquimbo	■■■■■■■■■■■■■■■■■■■■■■■■■■■■■■□■□□□□■■■■■■■	777777777720771	50	H3	Pansusceptible
1260-2011-S-793	2011	Female	Santiago	■■■■■■■■■■■■■■■■■■■■■■■■■■■■■■□■□□□□■■■■■■■	777777777720771	50	H3	Pansusceptible
1261-2011-S-794	2011	Female	Santiago	■■■■■■■■■■■■■■■■■■■■■■■■■■■■■■□■□□□□■■■■■■■	777777777720771	50	H3	Pansusceptible
1265-2011-S-798	2011	Male	Iquique	■■■■■■■■■■■■■■■■■■■■■■■■■■■■■■□■□□□□■■■■■■■	777777777720771	50	H3	Pansusceptible
1281-2011-S-808	2011	Male	Santiago	■■■■■■■■■■■■■■■■■■■■■■■■■■■■■■□■□□□□■■■■■■■	777777777720771	50	H3	Pansusceptible
1326-2011-S-838	2011	Male	Santiago	■■■■■■■■■■■■■■■■■■■■■■■■■■■■■■□■□□□□■■■■■■■	777777777720771	50	H3	MDR (INH+RF)
1359-2011-S-861	2011	Male	Santiago	■■■■■■■■■■■■■■■■■■■■■■■■■■■■■■□■□□□□■■■■■■■	777777777720771	50	H3	Pansusceptible
139-2012-S-121	2012	Male	Santiago	■■■■■■■■■■■■■■■■■■■■■■■■■■■■■■□■□□□□■■■■■■■	777777777720771	50	H3	R (INH)
1397-2011-S-889	2011	Male	Santiago	■■■■■■■■■■■■■■■■■■■■■■■■■■■■■■□■□□□□■■■■■■■	777777777720771	50	H3	Pansusceptible
1399-2011-S-891	2011	Male	Coquimbo	■■■■■■■■■■■■■■■■■■■■■■■■■■■■■■□■□□□□■■■■■■■	777777777720771	50	H3	Pansusceptible
1438-2011-S-923	2011	Male	Santiago	■■■■■■■■■■■■■■■■■■■■■■■■■■■■■■□■□□□□■■■■■■■	777777777720771	50	H3	Pansusceptible
1457-2011-S-939	2011	Male	Arica	■■■■■■■■■■■■■■■■■■■■■■■■■■■■■■□■□□□□■■■■■■■	777777777720771	50	H3	Pansusceptible
1746-2011-S-1161	2011	Male	Rancagua	■■■■■■■■■■■■■■■■■■■■■■■■■■■■■■□■□□□□■■■■■■■	777777777720771	50	H3	Pansusceptible
1942-2011-S-1351	2011	Female	Santiago	■■■■■■■■■■■■■■■■■■■■■■■■■■■■■■□■□□□□■■■■■■■	777777777720771	50	H3	Pansusceptible
1948-2011-S-1353	2011	Female	Copiapo	■■■■■■■■■■■■■■■■■■■■■■■■■■■■■■□■□□□□■■■■■■■	777777777720771	50	H3	Pansusceptible
496-2012-S-341	2012	Male	Santiago	■■■■■■■■■■■■■■■■■■■■■■■■■■■■■■□■□□□□■■■■■■■	777777777720771	50	H3	Pansusceptible
702-2012-S-466	2012	Male	Antofagasta	■■■■■■■■■■■■■■■■■■■■■■■■■■■■■■□■□□□□■■■■■■■	777777777720771	50	H3	Pansusceptible
708-2012-S-472	2012	Female	Rancagua	■■■■■■■■■■■■■■■■■■■■■■■■■■■■■■□■□□□□■■■■■■■	777777777720771	50	H3	Pansusceptible
745-2012-S-494	2012	Male	Santiago	■■■■■■■■■■■■■■■■■■■■■■■■■■■■■■□■□□□□■■■■■■■	777777777720771	50	H3	Pansusceptible
836-2011-S-510	2011	Male	Arica	■■■■■■■■■■■■■■■■■■■■■■■■■■■■■■□■□□□□■■■■■■■	777777777720771	50	H3	Pansusceptible
845-2011-S-514	2011	Male	Valparaiso	■■■■■■■■■■■■■■■■■■■■■■■■■■■■■■□■□□□□■■■■■■■	777777777720771	50	H3	Pansusceptible
860-2011-S-526	2011	Male	Santiago	■■■■■■■■■■■■■■■■■■■■■■■■■■■■■■□■□□□□■■■■■■■	777777777720771	50	H3	Pansusceptible
878-2012-S-567	2012	Female	Rancagua	■■■■■■■■■■■■■■■■■■■■■■■■■■■■■■□■□□□□■■■■■■■	777777777720771	50	H3	R (SM)
968-2011-S-593	2011	Male	Santiago	■■■■■■■■■■■■■■■■■■■■■■■■■■■■■■□■□□□□■■■■■■■	777777777720771	50	H3	R (INH)
98-2012-S-87	2012	Female	Viña del Mar	■■■■■■■■■■■■■■■■■■■■■■■■■■■■■■□■□□□□■■■■■■■	777777777720771	50	H3	Pansusceptible
1009-2011-S-625	2011	Male	Talcahuano	■■■■■■■■■■■■■■■■■■■■■■■■■■■■■■■■□□□□■■■□□□□	777777777760700	51	T1	Pansusceptible
135-2012-S-117	2012	Male	Santiago	■■■■■■■■■■■■■■■■■■■■■■■■■■■■■■■■□□□□■■■□□□□	777777777760700	51	T1	Pansusceptible
1863-2011-S-1288	2011	Female	Santiago	■■■■■■■■■■■■■■■■■■■■■■■■■■■■■■■■□□□□■■■□□□□	777777777760700	51	T1	Pansusceptible
1378-2011-S-878	2011	Male	Concepcion	■■■■■■■■■■■■■■■■■■■■■■■■■■■■■■■■□□□□■■■□■■■	777777777760731	52	T2	Pansusceptible
263-2012-S-204	2012	Female	Santiago	■■■■■■■■■■■■■■■■■■■■■■■■■■■■■■■■□□□□■■■□■■■	777777777760731	52	T2	Pansusceptible
1041-2012-S-660	2012	Male	Los Angeles	■■■■■■■■■■■■■■■■■■■■■■■■■■■■■■■■□□□□■■■■■■■	777777777760771	53	T1	Pansusceptible
1048-2011-S-654	2011	Male	Punta Arenas	■■■■■■■■■■■■■■■■■■■■■■■■■■■■■■■■□□□□■■■■■■■	777777777760771	53	T1	Pansusceptible
1130-2011-S-713	2011	Male	Santiago	■■■■■■■■■■■■■■■■■■■■■■■■■■■■■■■■□□□□■■■■■■■	777777777760771	53	T1	Pansusceptible
1149-2011-S-725	2011	Female	Osorno	■■■■■■■■■■■■■■■■■■■■■■■■■■■■■■■■□□□□■■■■■■■	777777777760771	53	T1	Pansusceptible
1170-2011-S-739	2011	Female	Santiago	■■■■■■■■■■■■■■■■■■■■■■■■■■■■■■■■□□□□■■■■■■■	777777777760771	53	T1	MDR (INH+RIF), SM
1173-2011-S-742	2011	Male	Valdivia	■■■■■■■■■■■■■■■■■■■■■■■■■■■■■■■■□□□□■■■■■■■	777777777760771	53	T1	Pansusceptible
1200-2011-S-759	2011	Female	Puerto Montt	■■■■■■■■■■■■■■■■■■■■■■■■■■■■■■■■□□□□■■■■■■■	777777777760771	53	T1	Pansusceptible
1228-2011-S-780	2011	Male	Santiago	■■■■■■■■■■■■■■■■■■■■■■■■■■■■■■■■□□□□■■■■■■■	777777777760771	53	T1	Pansusceptible
1299-2011-S-827	2011	Male	Temuco	■■■■■■■■■■■■■■■■■■■■■■■■■■■■■■■■□□□□■■■■■■■	777777777760771	53	T1	Pansusceptible
1325-2011-S-837	2011	Male	Talcahuano	■■■■■■■■■■■■■■■■■■■■■■■■■■■■■■■■□□□□■■■■■■■	777777777760771	53	T1	Pansusceptible
1328-2011-S-839	2011	Male	Antofagasta	■■■■■■■■■■■■■■■■■■■■■■■■■■■■■■■■□□□□■■■■■■■	777777777760771	53	T1	Pansusceptible
1358-2011-S-860	2011	Male	Los Angeles	■■■■■■■■■■■■■■■■■■■■■■■■■■■■■■■■□□□□■■■■■■■	777777777760771	53	T1	Pansusceptible
1361-2011-S-863	2011	Male	Valdivia	■■■■■■■■■■■■■■■■■■■■■■■■■■■■■■■■□□□□■■■■■■■	777777777760771	53	T1	Pansusceptible
1374-2011-S-874	2011	Female	Santiago	■■■■■■■■■■■■■■■■■■■■■■■■■■■■■■■■□□□□■■■■■■■	777777777760771	53	T1	Pansusceptible
1442-2011-S-927	2011	Female	Valparaiso	■■■■■■■■■■■■■■■■■■■■■■■■■■■■■■■■□□□□■■■■■■■	777777777760771	53	T1	Pansusceptible
1453-2011-S-936	2011	Female	Iquique	■■■■■■■■■■■■■■■■■■■■■■■■■■■■■■■■□□□□■■■■■■■	777777777760771	53	T1	Pansusceptible
1484-2011-S-962	2011	Female	Arica	■■■■■■■■■■■■■■■■■■■■■■■■■■■■■■■■□□□□■■■■■■■	777777777760771	53	T1	Pansusceptible
1588-2011-S-1041	2011	Female	Puerto Montt	■■■■■■■■■■■■■■■■■■■■■■■■■■■■■■■■□□□□■■■■■■■	777777777760771	53	T1	R (PZA)
1615-2011-S-1066	2011	Female	Santiago	■■■■■■■■■■■■■■■■■■■■■■■■■■■■■■■■□□□□■■■■■■■	777777777760771	53	T1	Pansusceptible
1628-2011-S-1078	2011	Female	Santiago	■■■■■■■■■■■■■■■■■■■■■■■■■■■■■■■■□□□□■■■■■■■	777777777760771	53	T1	Pansusceptible
1639-2011-S-1083	2011	Female	Osorno	■■■■■■■■■■■■■■■■■■■■■■■■■■■■■■■■□□□□■■■■■■■	777777777760771	53	T1	R (PZA)
1737-2011-S-1154	2011	Male	Rancagua	■■■■■■■■■■■■■■■■■■■■■■■■■■■■■■■■□□□□■■■■■■■	777777777760771	53	T1	Pansusceptible
1742-2011-S-1157	2011	Male	Rancagua	■■■■■■■■■■■■■■■■■■■■■■■■■■■■■■■■□□□□■■■■■■■	777777777760771	53	T1	Pansusceptible
1762-2011-S-1176	2011	Male	Rancagua	■■■■■■■■■■■■■■■■■■■■■■■■■■■■■■■■□□□□■■■■■■■	777777777760771	53	T1	Pansusceptible
189-2012-S-154	2012	Female	Ovalle	■■■■■■■■■■■■■■■■■■■■■■■■■■■■■■■■□□□□■■■■■■■	777777777760771	53	T1	MDR (INH+RF)
1905-2011-S-1322	2011	Male	Iquique	■■■■■■■■■■■■■■■■■■■■■■■■■■■■■■■■□□□□■■■■■■■	777777777760771	53	T1	Pansusceptible
1907-2011-S-1324	2011	Female	Iquique	■■■■■■■■■■■■■■■■■■■■■■■■■■■■■■■■□□□□■■■■■■■	777777777760771	53	T1	Pansusceptible
1917-2011-S-1332	2011	Female	Santiago	■■■■■■■■■■■■■■■■■■■■■■■■■■■■■■■■□□□□■■■■■■■	777777777760771	53	T1	Pansusceptible
1929-2011-S-1341	2011	Male	Rancagua	■■■■■■■■■■■■■■■■■■■■■■■■■■■■■■■■□□□□■■■■■■■	777777777760771	53	T1	Pansusceptible
1952-2011-S-1356	2011	Male	Osorno	■■■■■■■■■■■■■■■■■■■■■■■■■■■■■■■■□□□□■■■■■■■	777777777760771	53	T1	Pansusceptible
1955-2011-S-1359	2011	Female	Concepcion	■■■■■■■■■■■■■■■■■■■■■■■■■■■■■■■■□□□□■■■■■■■	777777777760771	53	T1	Pansusceptible
1987-2011-S-1382	2011	Female	Punta Arenas	■■■■■■■■■■■■■■■■■■■■■■■■■■■■■■■■□□□□■■■■■■■	777777777760771	53	T1	NA
247-2012-S-194	2012	Female	Santiago	■■■■■■■■■■■■■■■■■■■■■■■■■■■■■■■■□□□□■■■■■■■	777777777760771	53	T1	MDR (INH+RIF), SM
248-2012-S-195	2012	Male	Santiago	■■■■■■■■■■■■■■■■■■■■■■■■■■■■■■■■□□□□■■■■■■■	777777777760771	53	T1	Pansusceptible
270-2012-S-209	2012	Female	Castro	■■■■■■■■■■■■■■■■■■■■■■■■■■■■■■■■□□□□■■■■■■■	777777777760771	53	T1	Pansusceptible
271-2012-S-210	2012	Female	Castro	■■■■■■■■■■■■■■■■■■■■■■■■■■■■■■■■□□□□■■■■■■■	777777777760771	53	T1	Pansusceptible
289-2012-S-219	2012	Male	Santiago	■■■■■■■■■■■■■■■■■■■■■■■■■■■■■■■■□□□□■■■■■■■	777777777760771	53	T1	Pansusceptible
428-2012-S-302	2012	Male	Rancagua	■■■■■■■■■■■■■■■■■■■■■■■■■■■■■■■■□□□□■■■■■■■	777777777760771	53	T1	Pansusceptible
459-2012-S-320	2012	Male	Santiago	■■■■■■■■■■■■■■■■■■■■■■■■■■■■■■■■□□□□■■■■■■■	777777777760771	53	T1	R (SM)
479-2012-S-333	2012	Male	Santiago	■■■■■■■■■■■■■■■■■■■■■■■■■■■■■■■■□□□□■■■■■■■	777777777760771	53	T1	Pansusceptible
505-2012-S-347	2012	Female	Santiago	■■■■■■■■■■■■■■■■■■■■■■■■■■■■■■■■□□□□■■■■■■■	777777777760771	53	T1	Pansusceptible
596-2012-S-406	2012	Male	Los Angeles	■■■■■■■■■■■■■■■■■■■■■■■■■■■■■■■■□□□□■■■■■■■	777777777760771	53	T1	Pansusceptible
696-2012-S-462	2012	Female	Santiago	■■■■■■■■■■■■■■■■■■■■■■■■■■■■■■■■□□□□■■■■■■■	777777777760771	53	T1	R (INH+SM)
701-2012-S-465	2012	Male	Antofagasta	■■■■■■■■■■■■■■■■■■■■■■■■■■■■■■■■□□□□■■■■■■■	777777777760771	53	T1	Pansusceptible
841-2012-S-548	2011	Male	Los Angeles	■■■■■■■■■■■■■■■■■■■■■■■■■■■■■■■■□□□□■■■■■■■	777777777760771	53	T1	Pansusceptible
880-2011-S-534	2011	Male	Arica	■■■■■■■■■■■■■■■■■■■■■■■■■■■■■■■■□□□□■■■■■■■	777777777760771	53	T1	Pansusceptible
887-2011-S-540	2011	Male	Temuco	■■■■■■■■■■■■■■■■■■■■■■■■■■■■■■■■□□□□■■■■■■■	777777777760771	53	T1	Pansusceptible
913-2011-S-556	2011	Female	Santiago	■■■■■■■■■■■■■■■■■■■■■■■■■■■■■■■■□□□□■■■■■■■	777777777760771	53	T1	Pansusceptible
918-2012-S-587	2012	Male	Santiago	■■■■■■■■■■■■■■■■■■■■■■■■■■■■■■■■□□□□■■■■■■■	777777777760771	53	T1	Pansusceptible
919-2011-S-562	2011	Male	Coquimbo	■■■■■■■■■■■■■■■■■■■■■■■■■■■■■■■■□□□□■■■■■■■	777777777760771	53	T1	Pansusceptible
949-2011-S-582	2011	Male	Concepcion	■■■■■■■■■■■■■■■■■■■■■■■■■■■■■■■■□□□□■■■■■■■	777777777760771	53	T1	Pansusceptible
981-2011-S-605	2011	Male	Iquique	■■■■■■■■■■■■■■■■■■■■■■■■■■■■■■■■□□□□■■■■■■■	777777777760771	53	T1	Pansusceptible

998-2012-S-631	2012	Male	Los Angeles	■■■■■■■■■■■■■■■■■■■■■■■■■■■■■■■■□□□□■■■■■■■	777777777760771	53	T1	Pansusceptible
1275-2011-S-805	2011	Female	Iquique	■■■■■■■■■■■■■■■■■■■□■■□■■■■■■■■■■□□□□■■■■■■■	777777557760771	58	T5-Madrid2	Pansusceptible
1401-2011-S-893	2011	Male	Coquimbo	■■■■■■■■■■■■■■■■■■■□■■□■■■■■■■■■■□□□□■■■■■■■	777777557760771	58	T5-Madrid2	Pansusceptible
1403-2011-S-895	2011	Male	Coquimbo	■■■■■■■■■■■■■■■■■■■□■■□■■■■■■■■■■□□□□■■■■■■■	777777557760771	58	T5-Madrid2	Pansusceptible
1755-2011-S-1168	2011	Male	Rancagua	■■■■■■■■■■■■■■■■■■■□■■□■■■■■■■■■■□□□□■■■■■■■	777777557760771	58	T5-Madrid2	Pansusceptible
1213-2011-S-767	2011	Male	Santiago	■■■■■■■■■■■■■■■■■■■■□□□□■■■■■■■■□□□□■■■□■■■	777777607760731	60	LAM4	Pansusceptible
1232-2011-S-782	2011	Male	Santiago	■■■■■■■■■■■■■■■■■■■■□□□□■■■■■■■■□□□□■■■□■■■	777777607760731	60	LAM4	Pansusceptible
1416-2011-S-906	2011	Male	Santiago	■■■■■■■■■■■■■■■■■■■■□□□□■■■■■■■■□□□□■■■□■■■	777777607760731	60	LAM4	Pansusceptible
1494-2011-S-968	2011	Female	Valparaiso	■■■■■■■■■■■■■■■■■■■■□□□□■■■■■■■■□□□□■■■□■■■	777777607760731	60	LAM4	Pansusceptible
306-2012-S-233	2012	Male	Concepcion	■■■■■■■■■■■■■■■■■■■■□□□□■■■■■■■■□□□□■■■□■■■	777777607760731	60	LAM4	Pansusceptible
791-2012-S-519	2012	Female	Santiago	■■■■■■■■■■■■■■■■■■■■■■□□□■■■■■■■□□□□■■■■■■■	777777743760771	61	Cameroon	Pansusceptible
925-2011-S-569	2011	Female	Santiago	■■■■■■■■■■■■■■■■■■■■■■■■■□□□□□□■□□□□■■■□■■■	777777774020731	62	H1	Pansusceptible
1472-2011-S-949	2011	Female	Santiago	■■■■■■■■■■■■■■■■■■■■□□□□■■■■□■■■□□□□■■■■■■■	777777607560771	64	LAM6	Pansusceptible
1485-2011-S-963	2011	Male	Arica	■■■■■■■■■■■■■■■■■■■■□□□□■■■■□■■■□□□□■■■■■■■	777777607560771	64	LAM6	Pansusceptible
181-2012-S-146	2012	Male	Santiago	■■■■■■■■■■■■■■■■■■■■□□□□■■■■□■■■□□□□■■■■■■■	777777607560771	64	LAM6	Pansusceptible
182-2012-S-147	2012	Male	Santiago	■■■■■■■■■■■■■■■■■■■■□□□□■■■■□■■■□□□□■■■■■■■	777777607560771	64	LAM6	Pansusceptible
1903-2011-S-1320	2011	Male	Iquique	■■■■■■■■■■■■■■■■■■■■□□□□■■■■□■■■□□□□■■■■■■■	777777607560771	64	LAM6	Pansusceptible
224-2012-S-178	2012	Male	Santiago	■■■■■■■■■■■■■■■■■■■■□□□□■■■■□■■■□□□□■■■■■■■	777777607560771	64	LAM6	Pansusceptible
511-2012-S-350	2012	Male	Santiago	■■■■■■■■■■■■■■■■■■■■□□□□■■■■□■■■□□□□■■■■■■■	777777607560771	64	LAM6	R (SM)
971-2011-S-596	2011	Male	Castro	■■■■■■■■■■■■■■■■■■■■□□□□■■■■□■■■□□□□■■■■■■■	777777607560771	64	LAM6	Pansusceptible
1049-2011-S-655	2011	Male	Punta Arenas	■■■■■■■■■■■■■■■■■■■■■■■■■■■■■■■■□□□□■■■□□■■	777777777760711	78	T	R (SM)
1379-2011-S-879	2011	Male	Arica	■■■■■■■■■□■■■■■■■■■■□□□□■■■■■■■■□□□□■■■■■■■	777377607760771	81	LAM9	Pansusceptible
1269-2011-S-801	2011	Male	Iquique	■■■□□□□□□□□□□■■■■□■■■■■■■■■■■■■■□□□□■■■■■■■	700036777760771	91	X3	Pansusceptible
1273-2011-S-803	2011	Male	Iquique	■■■□□□□□□□□□□■■■■□■■■■■■■■■■■■■■□□□□■■■■■■■	700036777760771	91	X3	Pansusceptible
526-2012-S-358	2012	Female	Ovalle	■■■□□□□□□□□□□■■■■□■■■■■■■■■■■■■■□□□□■■■■■■■	700036777760771	91	X3	NA
737-2012-S-490	2012	Male	Los Angeles	■■■□□□□□□□□□□■■■■□■■■■■■■■■■■■■■□□□□■■■■■■■	700036777760771	91	X3	R (INH+SM)
976-2011-S-600	2011	Male	Iquique	■■■□□□□□□□□□□■■■■□■■■■■■■■■■■■■■□□□□■■■■■■■	700036777760771	91	X3	Pansusceptible
1223-2011-S-776	2011	Female	Antofagasta	■■■■■■■■■■■■□■■■■■■■□□□□■■■■■■■■□□□□■■■■■■■	777737607760771	93	LAM5	Pansusceptible
1036-2012-S-656	2012	Male	Santiago	■■■■□■■■■■■■■■■■■■■■■■■■■■■■■□■□□□□■■■■■■■	757777777720771	99	H3	Pansusceptible
1393-2011-S-887	2011	Female	Osorno	■■■■□■■■■■■■■■■■■■■■■■■■■■■■■□■□□□□■■■■■■■	757777777720771	99	H3	Pansusceptible
881-2011-S-535	2011	Male	Arica	■■■■■■■■□□□■■■□■■■■■□□□□■■■■■■■■□□□□■■■■■■■	776167607760771	111	LAM3	Pansusceptible
936-2011-S-572	2011	Female	Puerto Montt	■■■■■■■■■■■■■■□■■■■■■■■■■■■■■■■■□□□□■■■■■■■	777767777760771	118	T1	Pansusceptible
1928-2011-S-1339	2011	Female	Rancagua	■■■■■■■■■■■■■■■■■□■■■■■■■■■■■■■■□□□□■■■■■■■	777776777760771	119	X1	Pansusceptible
937-2011-S-573	2011	Male	Los Angeles	■■■■■■■■■■■■■■■■■■■■■■■■■■■■■■□□□□□□■■■■■■■	777777777700771	124	Unknown	Pansusceptible
1287-2011-S-814	2011	Male	Valparaiso	■■■■■■■■□□□■■■■■■■■■□□□□■■■■■■■■□□□□■■■□■■■	776177607760731	130	LAM3	Pansusceptible
1381-2011-S-881	2011	Male	Concepcion	■■■■■■■■□□□■■■■■■■■■□□□□■■■■■■■■□□□□■■■□■■■	776177607760731	130	LAM3	Pansusceptible
1003-2011-S-621	2011	Male	Talca	■■■■■■■■■■■■■■■■■□■■■■■■■■■■■■■■□□□□■■□□□□■	777776777760601	137	X2	R (INH)
1274-2011-S-804	2011	Female	Iquique	■■■■■■■■■■■■■■■■■□■■■■■■■■■■■■■■□□□□■■□□□□■	777776777760601	137	X2	Pansusceptible
1980-2011-S-1376	2011	Male	Valparaiso	■■■■■■■■■■■■■■■■■□■■■■■■■■■■■■■■□□□□■■□□□□■	777776777760601	137	X2	Pansusceptible
205-2012-S-167	2012	Male	Rancagua	■■■■□■■■■■■■■■■■■■■■■■■■■■■■■■■□□□□■■■■■■■	757777777760771	154	T1	Pansusceptible
1032-2011-S-638	2011	Male	Concepcion	■■■■■■■■■■■■■■■■■■■■□□□□■■■■■■■■□□□□■■□■■■■	777777607760671	162	LAM9	R (PZA)
694-2012-S-460	2012	Male	Santiago	■■■■■■■■■■■■■■■■■■■■□□□□■■■■■■■■□□□□■■■□□□□	777777607760700	163	LAM4	Pansusceptible
245-2012-S-192	2012	Female	Santiago	■■□■■■■■■■■■■■■■■■■■■■■■■■■■■■□■□□□□■■■■■■■	677777777720771	180	H3	R (INH+SM)
269-2012-S-208	2012	Male	Santiago	■■□■■■■■■■■■■■■■■■■■■■■■■■■■■■□■□□□□■■■■■■■	677777777720771	180	H3	Pansusceptible
1402-2011-S-894	2011	Female	Coquimbo	■■□■■■■■■■■■□■■■■■■■□□□□■■■■■■■■□□□□■■■□■■■	677737607760731	194	LAM2	Pansusceptible
1898-2011-S-1316	2011	Female	Iquique	■■□■■■■■■■■■□■■■■■■■□□□□■■■■■■■■□□□□■■■□■■■	677737607760731	194	LAM2	Pansusceptible
1410-2011-S-901	2011	Female	Santiago	■■□■■■■■■■■■■■■■■■■■□□□□■■■■■□■■□□□□■■■■■■■	677777607660771	195	LAM1	Pansusceptible
1163-2011-S-734	2011	Male	Santiago	■■■■■■■■□□□■□■■■■■■■□□□□■■■■■■■■□□□□■■■■■■■	776137607760771	211	LAM3	Pansusceptible
1177-2011-S-745	2011	Male	Temuco	■■■■■■■■□□□■□■■■■■■■□□□□■■■■■■■■□□□□■■■■■■■	776137607760771	211	LAM3	Pansusceptible
1193-2011-S-755	2011	Female	Concepcion	■■■■■■■■□□□■□■■■■■■■□□□□■■■■■■■■□□□□■■■■■■■	776137607760771	211	LAM3	Pansusceptible
1297-2011-S-825	2011	Male	Temuco	■■■■■■■■□□□■□■■■■■■■□□□□■■■■■■■■□□□□■■■■■■■	776137607760771	211	LAM3	Pansusceptible
1368-2011-S-868	2011	Male	Temuco	■■■■■■■■□□□■□■■■■■■■□□□□■■■■■■■■□□□□■■■■■■■	776137607760771	211	LAM3	Pansusceptible
187-2012-S-152	2012	Male	Ovalle	■■■■■■■■□□□■□■■■■■■■□□□□■■■■■■■■□□□□■■■■■■■	776137607760771	211	LAM3	Pansusceptible
1959-2011-S-1363	2011	Male	Concepcion	■■■■■■■■□□□■□■■■■■■■□□□□■■■■■■■■□□□□■■■■■■■	776137607760771	211	LAM3	Pansusceptible
704-2012-S-468	2012	Male	Rancagua	■■■■■■■■□□□■□■■■■■■■□□□□■■■■■■■■□□□□■■■■■■■	776137607760771	211	LAM3	Pansusceptible
922-2011-S-566	2011	Male	Santiago	■■■■■■■■□□□■□■■■■■■■□□□□■■■■■■■■□□□□■■■■■■■	776137607760771	211	LAM3	Pansusceptible
927-2011-S-570	2011	Female	Santiago	■■■■■■■■□□□■□■■■■■■■□□□□■■■■■■■■□□□□■■■■■■■	776137607760771	211	LAM3	Pansusceptible
930-2012-S-598	2012	Male	Santiago	■■■■■■■■□□□■□■■■■■■■□□□□■■■■■■■■□□□□■■■■■■■	776137607760771	211	LAM3	Pansusceptible
938-2011-S-574	2011	Male	Valdivia	■■■■■■■■□□□■□■■■■■■■□□□□■■■■■■■■□□□□■■■■■■■	776137607760771	211	LAM3	Pansusceptible
1897-2011-S-1315	2011	Male	Iquique	■■■■■■■■■■■■■■■■□□□□□■■■■■■■□■■■□□□□■■■■■■■	777774077560771	222	Unknown	Pansusceptible
2015-2011-S-1402	2011	Male	Rancagua	■■■■■■■■■■■■■■■■□□□□□■■■■■■■□■■■□□□□■■■■■■■	777774077560771	222	Unknown	Pansusceptible
510-2012-S-349	2012	Male	Santiago	■■■■■■■■■■■■■■■■□□□□□■■■■■■■□■■■□□□□■■■■■■■	777774077560771	222	Unknown	R (SM)
947-2011-S-580	2011	Male	Concepcion	■■■■■■■■■■■■■■■■□□□□□■■■■■■■□■■■□□□□■■■■■■■	777774077560771	222	Unknown	Pansusceptible
839-2012-S-546	2011	Male	Los Angeles	■■■■■■■■■■■■■■■■■■■■■■■■■■■■■■□□□□□□□□■■■■■	777777777700171	238	Unknown	R (SM)
1640-2011-S-1084	2011	Female	Osorno	■■■■■■■■■■■■■■■■■■■■■■■■■■■■■■■■□□□□□□□□■■■	777777777760031	239	T2	Pansusceptible
1134-2011-S-715	2011	Male	Copiapo	■■■■■■■■■■■■■■■■■■■■■■■■■■■■■■■■□□□□■□□□□■■	777777777760411	241	T1	Pansusceptible
985-2011-S-608	2011	Female	Temuco	■■■■■■■■■■■■■■■■■■■■■■■■■■■■■■■■□□□□■■□□□□■	777777777760601	244	T1	Pansusceptible
967-2011-S-592	2011	Male	Valparaiso	■■■■■■■■■■■■■■■■■■■■■■■■■■■■■■■■□□□□■■□■■■■	777777777760671	245	T1	Pansusceptible
1131-2011-S-714	2011	Male	Santiago	■■■■■■■■■■■■■■■■■■■■■□□□■□□□□□□■□□□□■■■■■■■	777777704020771	283	H1	Pansusceptible
1353-2011-S-856	2011	Male	Santiago	■■■■■■■■■■■■■■■■■■■■■□□□■□□□□□□■□□□□■■■■■■■	777777704020771	283	H1	Pansusceptible
1443-2011-S-928	2011	Male	Valparaiso	■■■■■■■■■■■■■■■■■■■■■□□□■□□□□□□■□□□□■■■■■■■	777777704020771	283	H1	MDR (INH+RF)
254-2012-S-198	2012	Female	Santiago	■■■■■■■■■■■■■■■■■■■■■□□□■□□□□□□■□□□□■■■■■■■	777777704020771	283	H1	Pansusceptible
290-2012-S-220	2012	Male	Santiago	■■■■■■■■■■■■■■■■■■■■■□□□■□□□□□□■□□□□■■■■■■■	777777704020771	283	H1	Pansusceptible
998-2011-S-618	2011	Male	Valparaiso	■■■■■■■■■■■■■■■■■■■■■□□□■□□□□□□■□□□□■■■■■■■	777777704020771	283	H1	Pansusceptible
180-2012-S-145	2012	Male	Santiago	■■■■■■■■■■■■■□■■■■■■■■■■■■■■■■■■□□□□■■■■■■■	777757777760771	393	T1	Pansusceptible
1337-2011-S-843	2011	Female	Santiago	■■■□□□■□□□□□□□□□■■□■■■□□■■■■■■■■□□□□■□□■■■■	704003347760471	430	T4-CEU1	Pansusceptible
288-2012-S-218	2012	Male	Santiago	■■■■■□□□□■■■■■■■■■■■■■■■■■■■■■■□□□□□□■■■■■■	760777777740371	434	T1	Pansusceptible
1341-2011-S-846	2011	Male	Antofagasta	■■■■■□□■■■■■■■■■■■■■□□□□■■■■■■■■□□□□■■■■■■■	763777607760771	435	LAM9	Pansusceptible
886-2011-S-539	2011	Female	Temuco	■■■■■■■■■■■■■■■■■□■■■■■■□□□□□□□□□□□□□□□□□□□	777776770000000	450	Unknown	Pansusceptible
1089-2011-S-682	2011	Male	Osorno	■■■■■■■■■■■■□■■■■■■■■□■■■■■■■■■■□□□□■■■■■■■	777737737760771	504	T3	Pansusceptible
1180-2011-S-748	2011	Male	Santiago	■■■■■■■■□□□■■■■■■■■■□□□□■■■■■■■■□□□□□■■■■■■	776177607760371	720	LAM3	Pansusceptible
1234-2011-S-784	2011	Female	Santiago	■■■■■■■■□□□■■■■■■■■■□□□□■■■■■■■■□□□□□■■■■■■	776177607760371	720	LAM3	Pansusceptible
1235-2011-S-785	2011	Female	Santiago	■■■■■■■■□□□■■■■■■■■■□□□□■■■■■■■■□□□□□■■■■■■	776177607760371	720	LAM3	Pansusceptible
1437-2011-S-922	2011	Male	Santiago	■■■■■■■■□□□■■■■■■■■■□□□□■■■■■■■■□□□□□■■■■■■	776177607760371	720	LAM3	Pansusceptible
344-2012-S-255	2012	Male	Punta Arenas	■■■■■■■■□□□■■■■■■■■■□□□□■■■■■■■■□□□□□■■■■■■	776177607760371	720	LAM3	Pansusceptible
562-2012-S-381	2012	Male	Santiago	■■■■■■■■□□□■■■■■■■■■□□□□■■■■■■■■□□□□□■■■■■■	776177607760371	720	LAM3	Pansusceptible
1320-2011-S-834	2011	Female	Copiapo	■□□■■■■■■■■■■■■■■■■■□□□□■■■■■■■■□□□□■■■■■■■	477777607760771	753	LAM1	Pansusceptible
1404-2011-S-896	2011	Male	Osorno	■■■■■■■■■■■■■■■■■■■■■■■■■■■■■■■■□□□□□□□■■■■	777777777760071	787	T1	Pansusceptible
2058-2011-S-1434	2011	Female	Santiago	■□□□■■■■■■■■■■■■■■■■■■■■■■■■■■■■□□□□■■■■■■■	437777777760771	798	T1	Pansusceptible
2082-2011-S-1450	2011	Female	Concepcion	■□□□■■■■■■■■■■■■■■■■■■■■■■■■■■■■□□□□■■■■■■■	437777777760771	798	T1	Pansusceptible
1960-2011-S-1364	2011	Male	Concepcion	■■■■■■■■■■■■■■■■■■□□□□□□□□□■■■■■□□□□■■■■■■■	777777000760771	880	T1	Pansusceptible
1895-2011-S-1313	2011	Male	Iquique	■■□■■■■■■■■■■■■■■■■■□□□□■■■■■■■□□□□□□□□□□■■	677777607740011	900	LAM	Pansusceptible
287-2012-S-217	2012	Male	Santiago	■■■■■■■■□□■■■■■■■■■■■■■■■■■■■■□■□□□□■■■■■■■	776377777720771	914	Unknown	Pansusceptible
1382-2011-S-882	2011	Male	Concepcion	■■■■■■■■■■■■■■■■■■■■■■■□□□□□□□□■□□□□■■□□■■■	777777760020611	948	H3	Pansusceptible
262-2012-S-203	2012	Female	Santiago	■■■■■■■■■■■■■■■■■■■■■■■□□□□□□□□■□□□□■■□□■■■	777777760020611	948	H3	R (SM)
1736-2011-S-1153	2011	Female	Talca	■■■■■■■■■■■□□■■■■■■■■■■■■■■■■■■■□□□□■■■■■■■	777637777760771	1073	T1	Pansusceptible
1020-2011-S-631	2011	Male	Temuco	■■■■■■■■■■■■■■■□■■■■■■■■■■■■■■■■□□□□■■■■■■■	777773777760771	1105	T1	Pansusceptible
695-2012-S-461	2012	Male	Santiago	■■□□□□■■■■■■■■■■■■■■■■■■■■■■■□■□□□□■■■■■■■	607777777720771	1116	H3	Pansusceptible
664-2012-S-442	2012	Female	Santiago	■■■■□■■□□□■■■■■■■■■■□□□□■■■■■■■■□□□□■■■□□□□	754377607760700	1220	LAM	Pansusceptible
2016-2011-S-1403	2011	Male	Talcahuano	■■■■■■■■■■■■■■■■□□□□□■■■■■■■□■■■□□□□■■■□■■■	777774077560731	1232	T2	Pansusceptible
1081-2011-S-675	2011	Male	Temuco	■■■■■■■■■■■■■□□□□□□□□□□□□□□□□■■■□□□□■■■■■■■	777740000160771	1259	T1	Pansusceptible
1727-2011-S-1150	2011	Male	Santiago	■■■■■■■■■■■■■■■■■■□■□□□□■■■■■■■■□□□□■■■■■■■	777777207760771	1277	LAM9	Pansusceptible
1768-2011-S-1182	2011	Male	Santiago	■■■■■■■■■■■■■■■■■■□■□□□□■■■■■■■■□□□□■■■■■■■	777777207760771	1277	LAM9	Pansusceptible
2036-2011-S-1415	2011	Female	Iquique	■■■■■■■■■■■■■■■■■■□■□□□□■■■■■■■■□□□□■■■■■■■	777777207760771	1277	LAM9	R (SM)
2064-2011-S-1438	2011	Male	Valparaiso	■■■■■■■■■■■■■■■■■■□■□□□□■■■■■■■■□□□□■■■■■■■	777777207760771	1277	LAM9	Pansusceptible
824-2012-S-538	2012	Male	Punta Arenas	■■■■■■■■■■■■■■■■■■□■□□□□■■■■■■■■□□□□■■■■■■■	777777207760771	1277	LAM9	R (SM+PZA)
861-2011-S-527	2011	Male	Santiago	■■■■■■■■■■■■■■■■■■□■□□□□■■■■■■■■□□□□■■■■■■■	777777207760771	1277	LAM9	Pansusceptible
1152-2011-S-727	2011	Female	Santiago	■■■□■■■■□□□■■■■■■■■■□□□□■■■■■■□□□□□□□□■■■■■	736177607700171	1476	AFRI_2	Pansusceptible

109-2012-S-97	2012	Male	Talcahuano	■■■□□□□■■■■■■■■■■■■■■■■■■■■■■■■■□□□□■■■■■■■	703777777760771	1560	T1	Pansusceptible
1377-2011-S-877	2011	Female	Concepcion	■■■□□□□■■■■■■■■■■■■■■■■■■■■■■■■■□□□□■■■■■■■	703777777760771	1560	T1	Pansusceptible
457-2012-S-318	2012	Female	Santiago	■■■■■■■■■■■■■■■■■■■■■■■■■■□■■■■■□□□□■■■■■■■	777777776760771	1626	T1	Pansusceptible
1074-2011-S-668	2011	Male	Santiago	■■■■■■■■□□□□■■■■■■■■□□□□■■■■■■■■□□□□■■■■■■■	776077607760771	1685	LAM3	Pansusceptible
1094-2011-S-685	2011	Female	Los Angeles	■■■■■■■■□□□□■■■■■■■■□□□□■■■■■■■■□□□□■■■■■■■	776077607760771	1685	LAM3	Pansusceptible
1716-2011-S-1143	2011	Male	Concepcion	■■■■■■■■□□□□■■■■■■■■□□□□■■■■■■■■□□□□■■■■■■■	776077607760771	1685	LAM3	Pansusceptible
191-2012-S-156	2012	Male	Antofagasta	■■■□□■■■■■■■■■■■■■■■□□□□■■■■■■■■□□□□■■■■■■■	717777607760771	1706	LAM9	Pansusceptible
1967-2011-S-1367	2011	Female	Santiago	■■■■■■■■■■■■□■■■■■■■□□□□■■■■■■■■□□□□■■■□■■■	777737607760731	1710	LAM	R (RIF)
1968-2011-S-1368	2011	Female	Santiago	■■■■■■■■■■■■□■■■■■■■□□□□■■■■■■■■□□□□■■■□■■■	777737607760731	1710	LAM	Pansusceptible
421-2012-S-296	2012	Male	Los Angeles	■■■■■■■■■■■■□■■■■■■■□□□□■■■■■■■■□□□□■■■□■■■	777737607760731	1710	LAM	Pansusceptible
826-2011-S-503	2011	Male	Temuco	■■■■■■■■■■■■□■■■■■■■□□□□■■■■■■■■□□□□■■■□■■■	777737607760731	1710	LAM	Pansusceptible
908-2011-S-551	2011	Male	Concepcion	■■■■■■■■□□□■■■■■■■■■□□□□■■■■■■■□□□□□■■■■■■■	776177607740771	1841	LAM3	Pansusceptible
1447-2011-S-932	2011	Male	Valparaiso	■■■■■■■■■■■■■■■■■■■■■□□□■■■■■■■■□□□□■■■□■■■	777777707760731	1890	T2	Pansusceptible
1227-2011-S-779	2011	Female	Talca	■■■■■■■■□□□■■■■■■■■■□□□□■■■□■■■■□□□□■■■■■■■	776177607360771	2014	LAM3	Pansusceptible
1956-2011-S-1360	2011	Male	Concepcion	■■■□■■■■□□□■■■■■■■■■□□□□■■■■■■■■□□□□■■■■■■■	736177607760771	2015	LAM3	Pansusceptible
850-2011-S-519	2011	Male	Santiago	■■■□■■■■□□□■■■■■■■■■□□□□■■■■■■■■□□□□■■■■■■■	736177607760771	2015	LAM3	Pansusceptible
2094-2011-S-1460	2011	Male	Concepcion	■■■■■■■■■■■■■■■■■■■■□□□□■□□□□□□■□□□□■■■■■■■	777777604020771	2028	Unknown	Pansusceptible
2093-2011-S-1459	2011	Male	Valparaiso	■■■■■■■■□□□□□■■■■■■■□□□□■■■■■■■■□□□□■■■■■■■	776037607760771	2045	LAM3	Pansusceptible
997-2011-S-617	2011	Male	Valparaiso	■■□■■■■■■■■■■■■■■■■■□□□□■■■■■■■■□□□□□■■■■■■	677777607760371	2054	LAM	Pansusceptible
1063-2011-S-664	2011	Male	Santiago	■■■■■■■■■■■■■■■■■■■■□□■■■■■■■■■■□□□□■■■■■■■	777777637760771	2070	T1	Pansusceptible
1070-2011-S-666	2011	Male	Santiago	■■■■■■■■■■■■■■■■■■■■□□■■■■■■■■■■□□□□■■■■■■■	777777637760771	2070	T1	Pansusceptible
1118-2011-S-703	2011	Male	Copiapo	■■■■■■■■■■■■■■■■■■■■□□■■■■■■■■■■□□□□■■■■■■■	777777637760771	2070	T1	Pansusceptible
1165-2011-S-735	2011	Female	Puerto Montt	■■■■■■■■■■■■■■■■■■■■□□■■■■■■■■■■□□□□■■■■■■■	777777637760771	2070	T1	Pansusceptible
1062-2011-S-663	2011	Male	Santiago	■■■■■■■■□□□■■■■■■■■■■□□□□□□□□□□□□□□□□□□□□□□	776177600000000	2353	Unknown	Pansusceptible
30-2012-S-26	2012	Male	Santiago	■■■■■■■■□□□■■■■■■■■■■□□□□□□□□□□□□□□□□□□□□□□	776177600000000	2353	Unknown	Pansusceptible
1278-2011-S-806	2011	Male	Iquique	■■■■■■■■■■■■■■■■■■■■■■■■■■■■■■■■□□□□□□■■■■■	777777777760171	2366	T1	Pansusceptible
1398-2011-S-890	2011	Male	Santiago	■■■■■■■■□□□■■■■■■■■■□□□□■■■■■■■■□□□□■■■□□■■	776177607760711	2402	LAM3	Pansusceptible
1182-2011-S-750	2011	Female	Santiago	■■□■□■■■■■■■■□■■■■■■□□□□■■■■■■■■□□□□■■■■■■■	657757607760771	2512	LAM1	Pansusceptible
1891-2011-S-1310	2011	Female	Concepcion	■□■■■■■■■■■■■■■■■■■■□□□□■■■■■■■■□□□□■■■■□■■	577777607760751	2745	LAM9	Pansusceptible
911-2011-S-554	2011	Male	Santiago	■□■■■■■■■■■■■■■■■■■■□□□□■■■■■■■■□□□□■■■■□■■	577777607760751	2745	LAM9	Pansusceptible
1452-2011-S-935	2011	Female	Iquique	■■■■■■■■■■■■■■■■■■■□□■■■■■■■■■□■□□□□■■■■■■■	777777477720771	2885	H3	Pansusceptible
777-2012-S-510	2012	Male	Castro	■■■■■■■■■■■■□■■■■■■■■■■■■■■■■■□□□□□□■■■■■■■	777737777700771	2911	Unknown	Pansusceptible
1209-2011-S-764	2011	Male	Santiago	□□□■■■■□□□■■■■■■■■■■□□□□■■■■■■■■□□□□■■■□□□□	074377607760700	2941	LAM	Pansusceptible
1459-2011-S-941	2011	Male	Arica	■■■■■■■■■■■■■■■■■■■■■■■■■■■■□■■■□□□□■■■□■■■	777777777560731	3056	T2	Pansusceptible
1949-2011-S-1354	2011	Male	Osorno	□□□□□□□□□□□□□□□□□□□□□□□■■■■■■■■■□□□□■■■■■■■	000000017760771	3127	Unknown	Pansusceptible
982-2011-S-606	2011	Male	Iquique	■■■■■■■■□□□■■■■■■■■■■■■■■■■■■■■□■□□□□■■■■■■■	776177777720771	3251	H3	Pansusceptible
137-2012-S-119	2012	Female	Santiago	■■■■■■■■□□□■■■■■■■■■□□□□■■■■■■■■□□□□■■■■■■□	776177607760770	3472	LAM3	Pansusceptible
666-2012-S-444	2012	Female	Santiago	■■■■■■■■□□□■■■■■■■■■□□□□■■■■■■■■□□□□■■□■■■■	776177607760671	3491	LAM3	Pansusceptible
1384-2011-S-884	2011	Female	Copiapo	■□□□■■■■■■■■■■■■■■■■■■■■■■■■■■■■□□□□■■■□■■■	437777777760731	3934	T2	Pansusceptible
1288-2011-S-815	2011	Female	Iquique	■■■□□□■■■■■■■■■■□□□□□■■■■■■■□■■■□□□□■■■■■■■	707774077560771	3965	T1	MDR (INH+RIF), PZA
1282-2011-S-809	2011	Male	Concepcion	■■■■□□□□■■■■■■■■□□□□□■■■■■■■□■■■□□□□■■■■■■■	741774077560771	3967	T1	Pansusceptible
1002-2011-S-620	2011	Male	Santiago	■■■□□□□■■■■■■■■■■□■■■■■■■■■■■■■■□□□□■■■■■■■	703776777760771	4014*	X1	Pansusceptible
1008-2011-S-624	2011	Female	Talcahuano	■■■□□□□■■■■■■■■■■□■■■■■■■■■■■■■■□□□□■■■■■■■	703776777760771	4014*	X1	Pansusceptible
1893-2011-S-1311	2011	Male	Concepcion	■■■□□□□■■■■■■■■■■□■■■■■■■■■■■■■■□□□□■■■■■■■	703776777760771	4014*	X1	Pansusceptible
1957-2011-S-1361	2011	Male	Concepcion	■■■□□□□■■■■■■■■■■□■■■■■■■■■■■■■■□□□□■■■■■■■	703776777760771	4014*	X1	Pansusceptible
1958-2011-S-1362	2011	Female	Concepcion	■■■□□□□■■■■■■■■■■□■■■■■■■■■■■■■■□□□□■■■■■■■	703776777760771	4014*	X1	Pansusceptible
2021-2011-S-1405	2011	Female	Concepcion	■■■□□□□■■■■■■■■■■□■■■■■■■■■■■■■■□□□□■■■■■■■	703776777760771	4014*	X1	Pansusceptible
964-2012-S-617	2012	Male	Coihaique	■■■□□□□■■■■■■■■■■□■■■■■■■■■■■■■■□□□□■■■■■■■	703776777760771	4014*	X1	Pansusceptible
1006-2011-S-623	2011	Male	Talca	■■■■■■■■□□□■■■■■■■■■■□□□□■■■■■■■■□□□□■■■■■■	776177703770371	4015*	Unknown	Pansusceptible
1010-2011-S-626	2011	Male	Talcahuano	■■■■■■■■□□□■■■■■■■■■■□□□□■■■■■■■■□□□□■■■■■■	776177703770371	4015*	Unknown	R (PZA)
1012-2011-S-628	2011	Male	Santiago	■■■■■■■■□□□■■■■■■■■■■□□□□■■■■■■■■□□□□■■■■■■	776177703770371	4015*	Unknown	Pansusceptible
1029-2011-S-635	2011	Male	Concepcion	■■■■■■■■□□□■■■■■■■■■■□□□□■■■■■■■■□□□□■■■■■■	776177703770371	4015*	Unknown	Pansusceptible
1030-2011-S-636	2011	Male	Concepcion	■■■■■■■■□□□■■■■■■■■■■□□□□■■■■■■■■□□□□■■■■■■	776177703770371	4015*	Unknown	R (INH+SM)
1077-2011-S-671	2011	Male	Temuco	■■■■■■■■□□□■■■■■■■■■■□□□□■■■■■■■■□□□□■■■■■■	776177703770371	4015*	Unknown	Pansusceptible
1150-2011-S-726	2011	Male	Santiago	■■■■■■■■□□□■■■■■■■■■■□□□□■■■■■■■■□□□□■■■■■■	776177703770371	4015*	Unknown	NA
1059-2011-S-660	2011	Female	Santiago	■■■■■■■■■■■■■■■■■■■■■■■■■□□□□■□□□□□□■■■■■■■	777777774100771	4016*	Unknown	R (PZA)
1067-2011-S-665	2011	Male	Talcahuano	■■■■■■■■■■■■■■■■■■■■■■■■■□□□□■□□□□□□■■■■■■■	777777774100771	4016*	Unknown	Pansusceptible
1181-2011-S-749	2011	Male	Santiago	■■■■■■■■■■■■□■■■■■■■□□■■■■■■■■■■□□□□■■■■■■■	777737637760771	4017*	T3	Pansusceptible
1191-2011-S-753	2011	Female	Osorno	■■■■■■■■■■■■□■■■■■■■□□■■■■■■■■■■□□□□■■■■■■■	777737637760771	4017*	T3	Pansusceptible
1463-2011-S-944	2011	Female	Santiago	■■■■■■■■■■■■□■■■■■■■□□■■■■■■■■■■□□□□■■■■■■■	777737637760771	4017*	T3	R (INH)
1194-2011-S-756	2011	Male	Concepcion	■■■■■■■■■□■■■■■■■■■■□□■■■■□□□□□□□□□□■■■■■■■	777377636000771	4018*	Unknown	Pansusceptible
1143-2011-S-720	2011	Male	Talcahuano	■■■■■■■■■□■■■■■■■■■■□□■■■■□□□□□□□□□□■■■■■■■	777377636000771	4018*	Unknown	Pansusceptible
1211-2011-S-766	2011	Male	Talca	■■■■■■■■□□□■■■■■■■■■□□□□■■■■■■□■□□□□■■■■■■■	776177607720771	4019*	H3	Pansusceptible
1245-2011-S-787	2011	Female	Osorno	■■■■■■■■■□■■■□□■■■■■■■■■■■■■■■■■□□□□■■□■■■■	777347777760671	4020*	T1	Pansusceptible
1262-2011-S-795	2011	Male	Arica	■■■■■■■■■■■■□■■■■■■■■■■■■■■■■■□■□□□□■■□■■■■	777737777720671	4021*	H3	Pansusceptible
1285-2011-S-812	2011	Female	Valparaiso	■■■■■■■■■■■■■■■■■■□■■■■□■□□□□□□■□□□□■■■■■■■	777777364020771	4022*	H1	Pansusceptible
1303-2011-S-828	2011	Female	Santiago	■■■■■■■■■■■■■■■■□□□□□■■■■■■■□■■■□□□□■■■□□■■	777774077560711	4023*	T1	Pansusceptible
1329-2011-S-840	2011	Male	Valdivia	■■□■■□□■■■■■■■■■■■■■■■■■■■■■■■■□■□□□□■■■■■■■	663777777720771	4024*	H3	Pansusceptible
1988-2011-S-1383	2011	Female	Ovalle	■■□■■□□■■■■■■■■■■■■■■■■■■■■■■■■□■□□□□■■■■■■■	663777777720771	4024*	H3	Pansusceptible
444-2012-S-310	2012	Female	Santiago	■■□■■□□■■■■■■■■■■■■■■■■■■■■■■■■□■□□□□■■■■■■■	663777777720771	4024*	H3	R (SM)
133-2012-S-116	2012	Male	Santiago	■■■■■■■■■■■■■■□□■■□■■■□□■■■■■■■■□□□□■□□■■■■	777763347760471	4025*	T4-CEU1	Pansusceptible
183-2012-S-148	2012	Male	Santiago	■■■■■■■■■■■■■■□□■■□■■■□□■■■■■■■■□□□□■□□■■■■	777763347760471	4025*	T4-CEU1	Pansusceptible
675-2012-S-448	2012	Male	Santiago	■■■■■■■■■■■■■■□□■■□■■■□□■■■■■■■■□□□□■□□■■■■	777763347760471	4025*	T4-CEU1	NA
923-2011-S-567	2011	Male	Santiago	■■■■■■■■■■■■■■□□■■□■■■□□■■■■■■■■□□□□■□□■■■■	777763347760471	4025*	T4-CEU1	Pansusceptible
1340-2011-S-845	2011	Female	Santiago	■■■■■□□■■■■■■■■■■■■■□□□□■■■■■■■■□□□□■■■□□□□	763777607760700	4026*	LAM4	Pansusceptible
727-2012-S-483	2012	Female	Santiago	■■■■■□□■■■■■■■■■■■■■□□□□■■■■■■■■□□□□■■■□□□□	763777607760700	4026*	LAM4	Pansusceptible
1418-2011-S-908	2011	Male	Santiago	■■■■■■□■□□□■■■■■■■■■□□□□■■■■■■■■□□□□■■■■■■■	772177607760771	4027*	LAM3	Pansusceptible
1421-2011-S-911	2011	Female	Santiago	■■■□□□□□□□□□□■■■■□■■■■■■■□□□■■■■□□□□■■■■■■■	700036774360771	4028*	X3	R (PZA)
1444-2011-S-929	2011	Male	Valparaiso	■■■■■■■■■■■■■■■■■■■■■□□■■■■■□■■■□□□□■■■■■■■	777777717560771	4029*	T1	Pansusceptible
1450-2011-S-933	2011	Female	Iquique	□□□□■■■■■■■■■■■■■■■■■□□□□□□□□□□□□□□□■■□□□■■	037777700000611	4030*	Unknown	Pansusceptible
979-2011-S-603	2011	Female	Iquique	□□□□■■■■■■■■■■■■■■■■■□□□□□□□□□□□□□□□■■□□□■■	037777700000611	4030*	Unknown	Pansusceptible
1489-2011-S-964	2011	Female	Santiago	■■■■■■■■■■■■■■■■□□□□□□■■■■■■□■■■□□□□■■■■■■■	777774037560771	4031*	T1	Pansusceptible
1491-2011-S-965	2011	Male	Santiago	■■■■■■■■■■■■■■■■□□□□□□■■■■■■□■■■□□□□■■■■■■■	777774037560771	4031*	T1	Pansusceptible
1653-2011-S-1095	2011	Female	Valparaiso	■■■■■■■■□□□□■■■■■■■■□□□□■■□□□■■■□□□□■■■■■■■	776077606160771	4032*	LAM3	Pansusceptible
1726-2011-S-1149	2011	Male	Santiago	■■■■■■■■□□□□■■■■■■■■□□□□■■□□□■■■□□□□■■■■■■■	776077606160771	4032*	LAM3	Pansusceptible
186-2012-S-151	2012	Male	Santiago	■■■■■■■■□□□■□■■■■■■■□□□□■■■■■■■■□□□□□■■■■■■	776137607760371	4033*	LAM3	Pansusceptible
1906-2011-S-1323	2011	Female	Iquique	■■■■■■■■□□□■■■■■■■■■□□□□■■■■■■■■■□□□□■■■■■■■	776177417760771	4034*	T1	Pansusceptible
1908-2011-S-1325	2011	Female	Iquique	■■■■■■■■□□□■■■■■■■■■□□□□■■■■■■■■■□□□□■■■■■■■	776177417760771	4034*	T1	Pansusceptible
2037-2011-S-1416	2011	Male	Iquique	■■□□□□□□■■■■■■■■■■■■■■■■■■■■■■■■□□□□■■■■■■■	601777777760771	4035*	T1	Pansusceptible
2070-2011-S-1443	2011	Male	Talca	■■□□□□□□■■■■■■■■■■■■■■■■■■■■■■■■□□□□■■■■■■■	601777777760771	4035*	T1	Pansusceptible
642-2012-S-429	2012	Male	Santiago	■■■■■■■■■■■■■■■■■■■■□□□□■■■□□■■■□□□□■■■□■■■	777777607160731	4036*	LAM4	Pansusceptible
966-2011-S-591	2011	Male	Talcahuano	■■■■■■■■■■■■■■■■■■■■■□■■■□□□□□□■□□□□■■■■■■■	777777734020771	4037*	H1	Pansusceptible
1005-2011-S-622	2011	Female	Talca	■■■■■■■■□□□■■■■■■■■■□□□□■□■■■■■■□□□□■■■□■■■	776177605760731	Or01	LAM3	NA
1045-2011-S-651	2011	Male	Santiago	■■■■■■■■□□□■■■■■■■■■□□□□■■■□□■■■□□□□■■■■■■■	776177607160771	Or02	LAM3	Pansusceptible
1083-2011-S-677	2011	Male	Castro	■■■□□□□□■■■■■■■■■■■■■■■■■■■■■■■■□□□□■■■■■■■	701777777760771	Or03	T1	Pansusceptible
1110-2011-S-697	2011	Male	Puerto Montt	■■■■□□□□□□□□□□□□■■■■□□□□■■■■■■■■□□□□■■■■■■■	740003607760771	Or04	LAM3	Pansusceptible
1122-2011-S-707	2011	Male	Santiago	■■■■■■■■■■■■■■■■■■■■□□■■■■■■■■■□□□□□■■■■■■■	777777637740771	Or05	T1	Pansusceptible
1161-2011-S-732	2011	Female	Santiago	■■■■■■■■■■□□■■■■■■■■■■■■■□□□□□□■□□□□■■■■■■■	777477774020771	Or06	H1	Pansusceptible
1153-2011-S-728	2011	Female	Santiago	□■□■■■□□■□□□□□□■□□■■□□□□■■□■■■■■□□□□■■■□■□□	271004606760720	Or07	LAM2	R (PZA)
1192-2011-S-754	2011	Female	Osorno	■■■□■■■■■□□■■■■■■■■■□□□□■■■■■■■■□□□□■■■■■■■	737177607760771	Or08	LAM9	Pansusceptible
1214-2011-S-768	2011	Male	Santiago	■■■■□□□□■■■■■■■■□□□□□□■■■■■■□■■■□□□□■■■■■■■	741774037560771	Or09	T1	Pansusceptible
1216-2011-S-770	2011	Female	Castro	■■■■■■■■□□□□■■■■■■■■□□□□■■■■■■■■□□□□■■■□■■■	776077607760731	Or10	LAM3	Pansusceptible
1220-2011-S-773	2011	Male	Coquimbo	■■■■■□□■■■■■■■■■■■■■□□□□■■■■■■■■□□□□■■■□■■■	763777607760731	Or11	LAM4	Pansusceptible
1335-2011-S-842	2011	Male	Santiago	■■■■■□□■■■■■■■□□□□■■□■■■■■■■■■■■□□□□■■■■■■■	763760677760771	Or12	T1	Pansusceptible
1375-2011-S-875	2011	Female	Castro	■■■□□□□■■■■■■■■■□■■■■■■■■■■■■■■■■□□□□■■■■■■■	703775777760771	Or13	T1	Pansusceptible
1376-2011-S-876	2011	Female	Concepcion	■■■■■■■■■□□■■■■■■■■■□□□□■■■■■■■■□□□□□■■■■■■	777177607760371	Or14	LAM9	Pansusceptible
138-2012-S-120	2012	Male	Santiago	■■■■■□□■■■■■■■□□□□■■□□□□■■■■■■■■□□□□■■■■■■■	763760607760771	Or15	LAM9	Pansusceptible
1419-2011-S-909	2011	Female	Santiago	■■■■■■□■■□□□■□□■■□■■□□■■■■■■■■■■□□□□■■■□□□■	773046637760701	Or16	X1	Pansusceptible

1439-2011-S-924	2011	Female	Santiago	■■■■■■■■■■■■■■■■■■■■■□■□□□□□□□□□□□□□□□□□□□■	777777720000001	Or17	Unknown	Pansusceptible
1451-2011-S-934	2011	Male	Iquique	■■■■□■■■■■■■■■■■■■■■□□□□■■■■■■□□□□□□■■■■■■■	757777607700771	Or18	Unknown	Pansusceptible
1468-2011-S-946	2011	Female	Valparaiso	■■■■■■■■□□□■■■■■■■■■■□□□■■■■■■■■■□□□□■■■■■■■	776177617760771	Or19	T1	Pansusceptible
1469-2011-S-947	2011	Male	Calama	■■■■■■■■■■■■■■□■■■■■■□□□■■■■■■■■□□□□■□□■■■■	777767707760471	Or20	T1	Pansusceptible
149-2012-S-123	2012	Female	Santiago	■■■□□□□□□□□□□□■■■■□■■■■■■■■■■■■■□□□□■■■■■■■	700017377760771	Or21	T4	Pansusceptible
1492-2011-S-966	2011	Male	Santiago	■■■□□■□□■□□□■■■■■■■■■□■■■■■■■■■■□□□□■■■■■■■	711077737760771	Or22	T1	Pansusceptible
1495-2011-S-969	2011	Male	Valparaiso	■■■□□□□□□■■■■■■■■■■■■■■■■■■■■■■■□□□□■■□■■■■	700777777760671	Or23	T1	Pansusceptible
1829-2011-S-1265	2011	Male	Santiago	■■■■■■■■■■■■□■■■□□□□□■■■■■■■□□■■□□□□■■■■■■■	777734077460771	Or24	T3	Pansusceptible
1830-2011-S-1266	2011	Male	Santiago	■■■■■■■■□□□■■■■■■□□■□□□□■■■■■■■■□□□□■■■■■■■	776176207760771	Or25	LAM3	Pansusceptible
1837-2011-S-1272	2011	Female	Santiago	■■■■□■■■■■■■■■■■■■■■■■■■□□□□■□■□□□□■■■■■■■	757777774120771	Or26	H3	Pansusceptible
185-2012-S-150	2012	Female	Santiago	■■□■■■■■□□□■□■■■■■■■□□□□■■■■■■■■□□□□■■■■■■■	676137607760771	Or27	LAM3	Pansusceptible
1944-2011-S-1352	2011	Male	Talcahuano	■■□■■■■■■■■■■■■■■■■□□□□■■■■■■■■■□□□□■■■■■■■	677777417760771	Or28	T1	Pansusceptible
1951-2011-S-1355	2011	Female	Osorno	□□□□□□□□□□□□□□□□□□□□□□□■□□□□□□□■□□□□■■■■■■■	000000010020771	Or29	H3	Pansusceptible
1953-2011-S-1357	2011	Female	Osorno	■■■■■■■■□□□■■■■■■■■□□□□■■■■■■■■■□□□□■■■□■■■	776177417760731	Or30	T2	Pansusceptible
1954-2011-S-1358	2011	Male	Osorno	■□■■■■■■■■■■■■■■■■■□□□□■■■■■■■■■□□□□■■■■□■■	577777417760751	Or31	T1	Pansusceptible
1981-2011-S-1377	2011	Male	Valparaiso	■■■■■■■■■■■■■■■■■■■■■■■□■■■■□□■■□□□□■■■■■■□	777777767460770	Or32	T1	Pansusceptible
305-2012-S-232	2012	Male	Concepcion	■■■■■■■■■■■■■■■□□□□□□□□■■■■■□■■■□□□□■■■■■■■	777770017560771	Or33	T1	Pansusceptible
372-2012-S-273	2012	Male	Coquimbo	■■■□■□□■■■■■■■■■■■□■□□□□■■■■■■■■□□□□■■■■■■■	723777207760771	Or34	LAM9	R (SM)
38-2012-S-32	2012	Female	Coquimbo	■□■■□■■■■■■■□■□■■■■■□□■■■■■■■■■□□□□□■■■■■■■	557727637740771	Or35	T3	Pansusceptible
39-2012-S-33	2012	Female	Concepcion	■□□□□□□□■■■■■■□■■■■■□□■■■■■■■■■□□□□□■■■■■■■	401767637740771	Or36	T1	Pansusceptible
50-2012-S-43	2012	Male	Santiago	■■■■■■■■■■■■■■□■■■■■□□■■■■■■■■□□□□□□■■■■■■■	777767637700771	Or37	Unknown	Pansusceptible
61-2012-S-53	2012	Female	Concepcion	■□■■■■■■□□□■□■□■■■■■□□□□■■■■■■■□□□□□■■■■■■■	576127607740771	Or38	LAM3	Pansusceptible
63-2012-S-55	2012	Male	Iquique	■■■■■■■■■■■■■■□■■■■■□□■■■■■■■■■□□□□□■■□□□■■	777767637740611	Or39	T1	Pansusceptible
64-2012-S-56	2012	Male	Puerto Montt	■□■■□■■■■■■■■■□■■■□■□□□□■■■■■■■□□□□□■■■■■■■	557767207740771	Or40	LAM9	Pansusceptible
66-2012-S-58	2012	Female	Valparaiso	■■□■■■■■□□□■■■□■■■■■□□□□■■■■■■■□□□□□■■■■■■■	676167607740771	Or41	LAM3	Pansusceptible
67-2012-S-59	2012	Male	Valparaiso	■□■■■■■■■■■■■■□■■■■■□□■■■■■■■■■□□□□□■■□□□□■	577767637740601	Or42	T1	Pansusceptible
676-2012-S-449	2012	Male	Santiago	■■□■■□□■■■■■■■■■■■■■■■■■■■■□■■□■□□□□■■■■■■■	663777777320771	Or43	H3	Pansusceptible
68-2012-S-60	2012	Male	Valparaiso	■■■■■■■■■■■■■■□■□□□□□□■■■■■■□■■□□□□□■■■■■■■	777764037540771	Or44	T1	Pansusceptible
883-2011-S-536	2011	Male	Arica	■■■■■■■■□□□■■■■■■■■■□□■■■■■■■■□■□□□□■■■■■■■	776177637720771	Or45	H3	Pansusceptible
894-2011-S-547	2011	Male	Antofagasta	■■■■■■■■■■■■■■■■■■■■□□■■■■■■■■□□□□□□■■■■■■■	777777637700771	Or46	Unknown	Pansusceptible
924-2011-S-568	2011	Male	Santiago	■□□□□■■■□□□□■■■■■■■■■□□□□■■■■■■■■□□□□■■■■■■	416077703770371	Or47	Unknown	Pansusceptible
977-2011-S-601	2011	Female	Iquique	■■■■■■■■■■■■□■■■■■■■□□□□■□■■■■■■□□□□■■■■■■■	777737605760771	Or48	LAM5	Pansusceptible
978-2011-S-602	2011	Male	Iquique	■□■■■■■■□□□■■■■■■■■■□□□□■■■■■■■■□□□□■■■□■■■	576177607760731	Or49	LAM3	R (PZA)
984-2011-S-607	2011	Male	Talca	■■■■■■■■□□□□■■■■■■■■□□□□■■■■■■□■□□□□□■■■■■■	776077607720371	Or50	H3	Pansusceptible

* NEW SITs are followed by an asterisk (*) and highlighted in yellow; Orphan spoligotypes are highlighted in blue.
** Drug resistance: pansusceptible n=402; NA, not available n=6; MDR, multidrug resistant n=6; R, any resistance, n=44. Abbreviations for first line drugs: INH, Isoniazid; RIF, Rifampin; SM, Streptomycin; PZA, pyrazinamide.
